# Electrocatalytic Properties and DFT Studies of Pd-Based Catalysts Supported on Ceria/Onion-like Carbon for Isopropanol Oxidation in Alkaline Medium

**DOI:** 10.1021/acs.jpcc.5c03686

**Published:** 2025-07-23

**Authors:** Desalegn Nigatu Gemechu, Aderemi B. Haruna, Ahmed Mustefa Mohammed, Yedilfana Setarge Mekonnen, Kenneth I. Ozoemena

**Affiliations:** † Department of Chemistry, College of Natural and Computational Sciences, Addis Ababa University, P.O. Box 1176 Addis Ababa, Ethiopia; ‡ Molecular Science Institute, School of Chemistry, University of the Witwatersrand, Johannesburg 2050, South Africa; § Center for Environmental Science, College of Natural and Computational Sciences, Addis Ababa University, P.O. Box 1176 Addis Ababa, Ethiopia

## Abstract

This study investigated the electrocatalytic performance of Pd-based catalysts supported on onion-like carbon (OLC) for isopropanol (IPA) oxidation in alkaline media (a direct isopropanol fuel cells (DIFC)). The synthesized and evaluated catalysts included PdNi/OLC, Pd/CeO_2_/OLC, and PdNi/CeO_2_/OLC, all of which demonstrated performances comparable to that of commercial Pd/C. These samples are characterized by physicochemical analytical methods such as X-ray diffraction (XRD), field-emission scanning electron microscopy (FE-SEM) coupled with energy-dispersive X-ray (EDX), high resolution transmission electron microscopy (HRTEM), X-ray photoelectron spectroscopy (XPS). Electrochemical measurements were also carried out using cyclic voltammetry (CV), chronoamperometry (CA), linear sweep voltammetry (LSV) and electrochemical impedance spectroscopy (EIS). The CV tests and Tafel analysis results revealed that PdNi/CeO_2_/OLC exhibited the highest current response with an onset potential of −0.69 V and better kinetic activity of 136.9 mV/dec respectively. Chronoamperometric tests confirmed that PdNi/CeO_2_/OLC maintains superior stability and antipoisoning properties, achieving stable current densities over extended periods. EIS showed the lowest charge transfer resistance (*R*_ct_) for PdNi/CeO_2_/OLC, reflecting more efficient electron transfer. The Ni-containing catalysts also exhibit lower Tafel slopes (121.5 mV/dec for PdNi/OLC and 136.9 mV/dec for PdNi/CeO_2_/OLC), indicating improved reaction kinetics due to Ni. Density functional theory (DFT) calculations suggest that Ni incorporation into Pd enhances electron donation, which is enhanced by the CeO_2_ support. This synergy improves IPA adsorption and reaction kinetics, leading to enhanced electrocatalytic performance. These findings demonstrate that alloying Pd with Ni and supporting it on CeO_2_ significantly boosts electrocatalytic activity, stability, and mass transport in alcohol oxidation. The PdNi/CeO_2_/OLC catalyst is a promising candidate for DIFC applications, offering high efficiency, durability, and a low onset potential.

## Graphical abstract

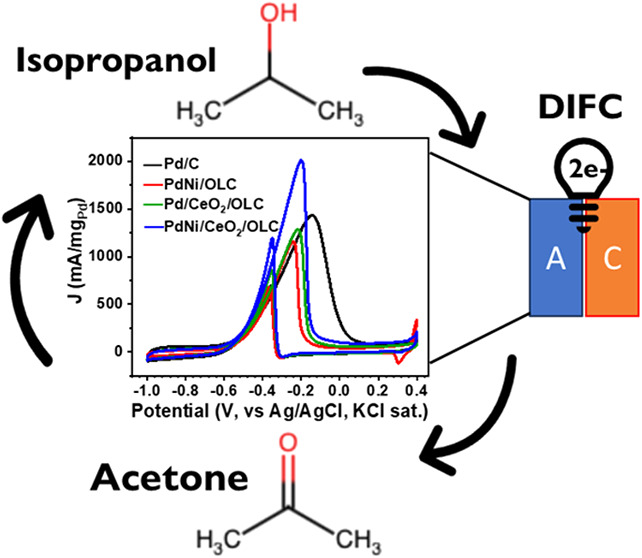

## Introduction

The increasing global demand for energy coupled with urgent environmental concerns presents significant challenges to our reliance on traditional fossil fuels. Shifting from current energy infrastructure to renewable, climate-neutral alternatives is essential for mitigating climate change. Given the complexity and time required to develop new energy systems, leveraging existing infrastructure is critical.^1–3^

Hydrogen technologies are emerging as promising solutions for the decarbonization of energy systems.^4–6^ Hydrogen can be produced through electrolysis of water using renewable energy sources, and subsequently converted back into electricity via fuel cells.^7,8^ However, the efficient storage of hydrogen gas remains a challenge due to its low volumetric energy density, even in its liquid form.^9^ Among the various storage methods, chemical storage of hydrogen using liquid organic hydrogen carriers (LOHCs) has gained considerable attention. LOHCs offer advantages such as improved safety, high efficiency, and zero CO_2_ emissions within the electrochemically relevant range, making them particularly attractive for secure hydrogen storage in sectors such as transportation and industry.^10–13^

Despite the potential of LOHCs, several challenges remain, particularly the energy-intensive dehydrogenation process and the requirement for additional hydrogen storage tanks. Isopropanol/acetone, as an LOHC, is specially promising in direct isopropanol fuel cells (DIFCs), which have emerged as attractive power sources for electronic devices because of their higher energy density, lower toxicity, and ease of handling compared to hydrogen gas.^14,15^ The electrooxidation product of isopropanol or isopropyl alcohol (IPA) is 2-propanone (acetone), which can be regenerated via catalytic or electrochemical hydrogenation, creating a closed-loop and efficient energy-storage cycle.^16–18^

DIFCs exhibit lower fuel crossover rates than conventional methanol and ethanol fuel cells, resulting in higher open-circuit voltages of up to 810 mV. The position of the OH group in secondary alcohols influences molecule adsorption on catalyst surfaces, altering oxidation pathways and reducing overpotential. Notably, IPA undergoes partial oxidation to acetone during fuel-cell operation, making it a viable alternative to methanol and ethanol. Its nontoxic nature and versatility as a solvent for disinfection have also increased interest in DIFCs.^16,19–21^

Research indicates that fuel cells using IPA achieve high open-circuit voltages, primarily because acetone is the main electrooxidation product with minimal CO_2_ generation.^20,22^ Operating DIFCs in alkaline media, rather than in acidic media, enhances electrode reaction kinetics and enables the use of cost-effective Pt-free catalysts, such as nickel, palladium and silver, thus improving overall performance.^23–26^

Carbon-supported Pd-based catalysts have gained significant attention owing to their diverse applications, notably as promising electrocatalysts for alkaline fuel cells.^27^ Pd is a highly effective and versatile catalyst for the electrooxidation of isopropanol, ethanol, glycerol, and formic acid, making it a strong candidate for various energy applications.^24,28,29^ Compared to Pt, Pd offers a more affordable alternative while maintaining excellent catalytic performance in alkaline media. Pd-based catalysts supported on carbon nanotubes (CNTs) significantly enhance electrooxidation activity, with palladium–niobium CNT bimetallic catalysts showing superior efficiency due to improved poisoning tolerance, lower onset potential, and faster charge transfer kinetics compared to monometallic Pd/CNTs or commercial Pd on carbon. Additionally, nanostructured Pd-based electrocatalysts in passive direct glycerol fuel cells exhibit strong catalytic activity, as demonstrated by the PdAu catalysts supported on vapor-grown carbon nanofibers (VGCNF), which achieved high current densities.^30–33^ Overall, Pd-based materials are promising clean energy technologies, offering improved efficiency, stability, and affordability compared to platinum-based alternatives.

Cerium oxide (CeO_2_), a rare-earth material valued for its abundance and cost-effectiveness, enhances the catalytic activities involving noble metals.^34,35^ Studies have shown that modifying Pd catalyst surfaces with ceria alters their electronic structure and thereby influencing adsorption energies and reaction configurations in alcohol oxidation.^36,37^ The robust oxygen storage capacity and redox properties of ceria, facilitated by Ce^4+^/Ce^3+^ cycles, are pivotal in oxidation reactions. Thus, ceria-supported catalysts with enhanced electronic structures effectively prevent catalyst deactivation, ensuring stable performance.^38,39^ Previous studies also indicate that nickel (Ni) exhibits a good performance in the oxidation of different alcohols.^40–42^

This study presents a novel approach for developing Pd-based electrocatalysts by incorporating Ni and CeO_2_ into OLC supports. The synthesis of PdNi/CeO_2_/OLC catalysts employs a straightforward coprecipitation method, which facilitates the formation of highly dispersed and stable active sites. This approach enhances the catalytic properties by leveraging the synergy between Pd, Ni, and CeO_2_, enhancing the catalyst electronic structure and stability for alcohol oxidation reactions. The incorporation of CeO_2_ helps tune the electronic properties of the Pd catalyst, while the addition of Ni further improves reaction kinetics, making the resulting catalyst a promising alternative to platinum-based materials for fuel cell applications.

This study focuses on the electrochemical oxidation of IPA using Pd-based electrocatalysts in an environmentally friendly potassium hydroxide, KOH electrolyte. The primary aim is to investigate the electrocatalytic oxidation of IPA on the synthesized catalysts, namely PdNi/OLC, Pd/CeO_2_/OLC, and PdNi/CeO_2_/OLC, using cyclic voltammetry (CV). The structural and morphological characteristics of these catalysts were analyzed using various techniques, including X-ray diffraction (XRD) and transmission electron microscopy (TEM), scanning electron microscope (SEM), X-ray photoelectron spectroscopy (XPS). Additionally, density functional theory (DFT) calculations were employed to explore their electronic properties and their influence on the catalytic performance of the synthesized electrocatalysts.

## Methodology

### Materials and Reagents

The OLC was obtained from NaBond. Platinum on activated carbon (10 wt %) and reagents (IPA 99%), including Potassium tetrachloropalladate­(II), K_2_PdCl_4_ (98%), Nickel chloride, NiCl_2_ (98%), ethylene glycol (99.8%), nafion solution, IPA (99.9%), Cerium­(III) nitrate hexahydrate, Ce­(NO_3_)_3_·6H_2_O (99.999%), and other analytical-grade chemicals like potassium hydroxide, KOH pellets, potassium chloride, KCl were purchased from Sigma-Aldrich. The water was prepared through an ultrapure purification system. All reagents were used without further purification.

### Synthesis of Pd-Based Electrocatalysts

#### Synthesis of CeO_2_/OLC Support (10 wt % CeO_2_)

A solution of Ce­(NO_3_)_3_·6H_2_O (76.43 mg) in H_2_O (25 mL) was prepared, into which OLC (270 mg) was added. The resulting mixture was stirred for 1 h and then sonicated for an additional 30 min. Subsequently, the pH was adjusted to 12 using a 2 M aqueous solution of KOH, and the resulting suspension was vigorously stirred for 2 h. The solid product was separated by filtration and washed with H_2_O until reaching a neutral pH. The product was then dried at 65 °C, followed by heating under air in a tube furnace at 250 °C for 3 h. Cooling to room temperature was carried out under a flow of Ar. The yield of OLC-CeO_2_ obtained was 298.4 mg. The synthesis procedure is shown in Figure 1.

**Figure 1:**
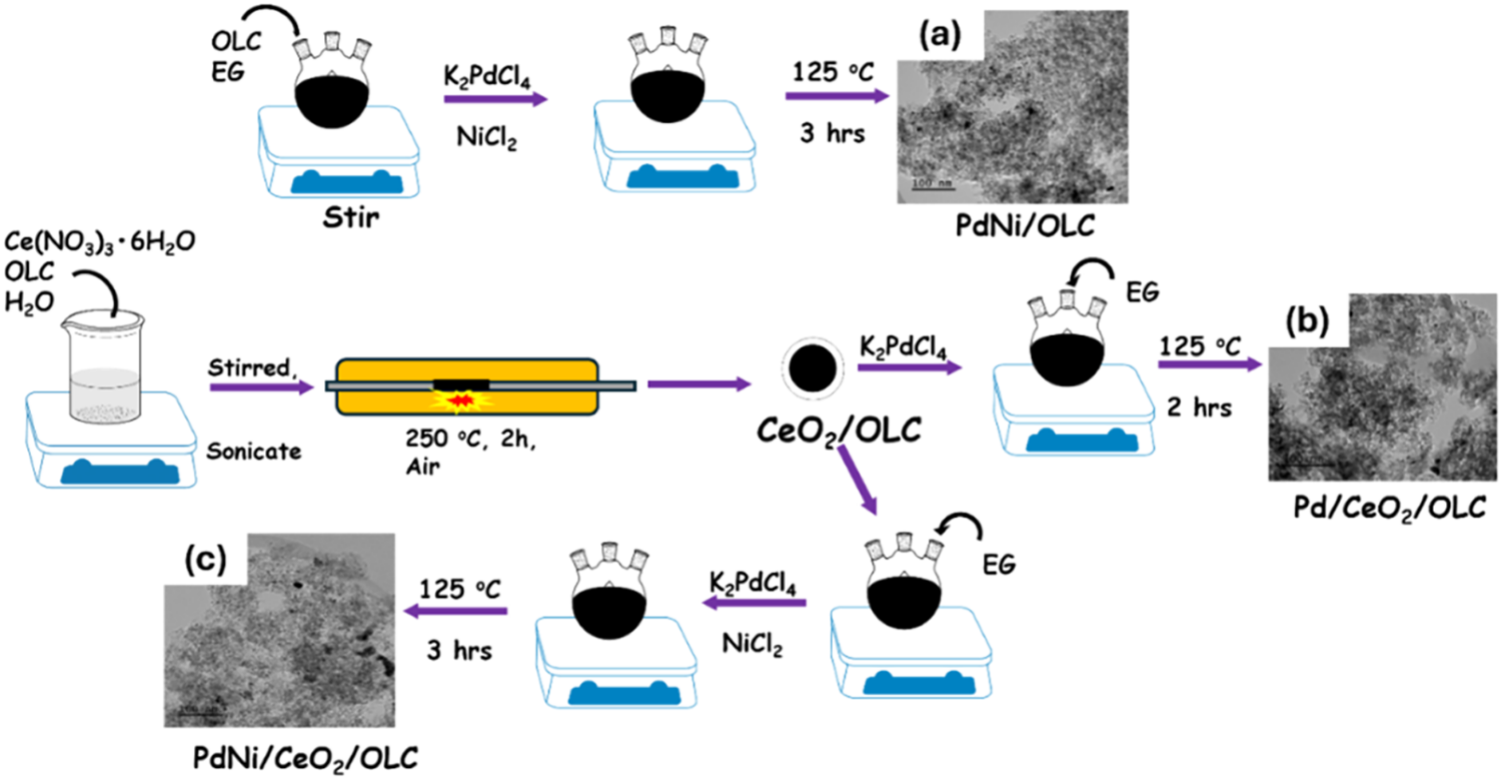
Synthesis pathways for the catalysts using wet impregnation and calcination method: (a) PdNi/OLC, (b)­Pd/CeO_2_/OLC, and (c) PdNi/CeO_2_/OLC.

#### Synthesis of Pd/Ni/OLC (10 wt % Pd, 10 wt % Ni)

The synthesis commenced with the ultrasonication of 120 mg of OLC in 100 mL of ethylene glycol within a three-neck round-bottomed flask for 1 h. Following this, a solution consisting of 10 mL of H_2_O, 10 mL of ethylene glycol, and 2 mL of 35% HCl, containing 46.93 mg of dissolved K_2_PdCl_4_, along with another solution containing 10 mL of ethylene glycol, 10 mL of water, and 33.2 mg of dissolved NiCl_2_, was added dropwise under stirring in a nitrogen stream to remove dissolved oxygen. After thorough stirring, an alkaline solution of NaOH (1 g) in 10 mL of H_2_O and 35 mL of ethylene glycol was introduced into the reactor, which was then heated at 125 °C for 3 h under a nitrogen atmosphere. Subsequently, the mixture was cooled to room temperature. The resulting solid product was filtered and washed with H_2_O until reaching a neutral pH. Finally, the product was dried in a vacuum oven at 40 °C, yielding 148.5 mg of the desired product. The synthesis procedure is shown in Figure 1(a).

#### Synthesis of Pd/CeO_2_/OLC (10 wt % Pd)

Figure 1(b) shows the synthesis procedure for Pd/CeO_2_/OLC. A suspension of CeO_2_–OLC (135 mg) in water (50 mL) was vigorously stirred for 30 min and sonicated for 30 min. To this mixture, a solution of K_2_PdCl_4_ (46.93 mg) in water (30 mL) was slowly added (1 mL min^–1^) under vigorous stirring, followed by the addition of an aqueous solution of 2.5 M KOH (1.0 mL). Subsequently, ethanol (20 mL) was added to the resulting mixture, which was then heated at 125 °C for 60 min. The desired product, Pd/CeO_2_–OLC, was filtered off, washed several times with distilled water until neutral pH was achieved, and finally dried under vacuum at 40 °C until a constant weight was obtained (Yield: 148.6 mg).

#### Synthesis of PdNi/CeO_2_/OLC (10 wt % Pd)

A suspension of OLC-CeO_2_ (200 mg) in water (50 mL) was vigorously stirred for 30 min and sonicated for 30 min. To this mixture, a solution of K_2_PdCl_4_ (46.93 mg) and NiCl_2_ (33.2 mg) in water (30 mL) was slowly added (1 mL min^–1^) under vigorous stirring, followed by the addition of an aqueous solution of 2.5 M KOH (1.0 mL). Subsequently, ethanol (20 mL) was added to the resulting mixture, which was then heated at 125 °C for 1 h. The desired product, PdNi/CeO_2_–OLC, was filtered off, washed several times with distilled water until neutral pH was achieved, and finally dried under vacuum at 40 °C until a constant weight was obtained (Yield: 151.9 mg). The synthesis procedure is shown in Figure 1(c).

### Material Characterization and Electrochemical Measurements

The structural features of the catalysts were determined through powder X-ray diffraction (PXRD), scanning electron microscopy (SEM), energy-dispersive X-ray spectroscopy (EDX), X-ray photoelectron spectroscopy (XPS) and high-resolution transmission electron microscopy (HRTEM) techniques. For electrochemical testing, a three-electrode setup was employed using a BioLogic electrochemical workstation. This setup included a glassy carbon electrode (GCE) with an area of 0.0707 cm^2^, platinum wire as the counter electrode, and an Ag/AgCl electrode as the reference electrode. To prepare the catalyst, 1 mg of catalyst powder was mixed with of 1 mL 70% IPA, 30% distilled water, and 0.25% Nafion, followed by sonication for 30 min. Subsequently, 10 μL of the mixture was cast on the surface of the cleaned GCE. The Pd loading of each electrode was maintained at 2.0 μg.

Measurements were carried out using cyclic voltammetry (CV), electrochemical impedance spectroscopy (EIS), and chronoamperometry (CA) with a workstation controlled by the EC-Lab software. Before starting each experiment, the cells were deaerated by bubbling nitrogen gas for 30 min. Electrochemical cleaning was performed to maintain a consistent surface structure, involving 20 voltammetry cycles ranging from −1.0 to 0.4 V at a scan rate of 100 mV/s against the Ag/AgCl reference. EIS was conducted at – 0.30 V vs Ag/AgCl by applying an alternating voltage of 5 mV from 200 kHz to 100 mHz. The electrolyte solution was 0.5 M KOH + 1.0 M IPA. All cyclic voltammograms (CVs) were recorded at a potential scan rate of 50 mV/s.

### Computational Model

In this work, four Pd-based model catalysts (Pd, Pd/Ni, Pd/CeO_2_, and Pd/Ni/CeO_2_) were designed and studied utilizing spin-polarized density functional theory (DFT) within the DMol3 module of Material Studio. Specifically, we focused on (111) surface of these catalysts as determined from the XRD analysis. DFT calculations were performed using the Perdew–Burke–Ernzerhof (PBE)^43^ exchange-correlation functional within the generalized gradient approximation (GGA), employing a double-numerical quality basis set with polarization functions (DNP).^44,45^ The Brillouin zone sampling was conducted using a (3 × 3 × 1) *k*-point grid based on the Monkhorst–Pack scheme.^46^ To take both computational accuracy and efficiency into account, the core treatment was used as density functional semicore pseudopotential (DSPP).^47^ The global orbital cutoff was set to 4.4 Å. A Grimme method was implemented to describe van der Waals interactions.^48^ The convergence criteria for geometry optimization and energy calculations were set at 1.0 × 10^–6^ Ha, 2.0 × 10^–6^ Ha, 0.004 Ha/Å, and 0.005 Å for the SCF tolerance, energy tolerance, maximum force, and maximum displacement, respectively.

The adsorption energy, *E*_ad_, which quantifies the strength of the interaction between the IPA (adsorbate) and the substrate (catalyst), was calculated using the following equation

1 $$E_{ad}=E_{slab+IPA}-E_{slab}-E_{IPA\left(g\right)}$$

where *E*_slab+IPA_ is the total energy of the slab surface after IPA adsorption, *E*_slab_ is the total energy of the surfaces, and *E*_IPA_ is the total energy of the isolated IPA molecule. To investigate the electronic interactions between IPA and slab, we employed the density of states (DOS) and d-band center of the electrocatalysts. The d band center (ε_d_) was calculated according to eq 2

2 $$\varepsilon_{d}=\frac{\int \rho EdE}{\int \rho dE}$$

where *E* is the energy with respect to the Fermi level and ρ is the electronic density of states. Adsorption locator tool from Material Studio module was used to identify the possible configuration of IPA adsorbed on the surfaces of the electrocatalysts. This tool uses Monte Carlo calculations to determine the most stable arrangement of the IPA on the electrocatalyst surface.

## Results and Discussion

### Materials Characterization

The PXRD patterns of the Pd-based catalysts (Figure 2) provide compelling evidence for the successful synthesis of the catalysts. Figure 2 shows the XRD patterns of the catalysts under study, revealing characteristic peaks corresponding to the crystallographic facets of Pd, Ni, CeO_2_, and C from OLC. Specifically, peaks observed at 2θ angles of 40, 47, 68, 82 and 86° correspond to the (111), (200), (220), (311) and (222) planes of Pd, respectively, indicating a face-centered cubic structure (JCPDS No. 46–1043).^28,49^ In addition, for Pd/CeO_2_/OLC and PdNi/CeO_2_/OLC, distinct peaks attributable to the face-centered cubic structure of CeO_2_ were identified at angles of 29, 33, 48, and 57° corresponding to reflections of (111), (200), (220) and (311).^50^ In the case of PdNi/OLC, two peaks are observed between 10° and 20° (2θ), which may correspond to the nickel oxide (NiO2_mp_25210) or β-Ni­(OH)_2_ (001) reflection.^51^

**Figure 2:**
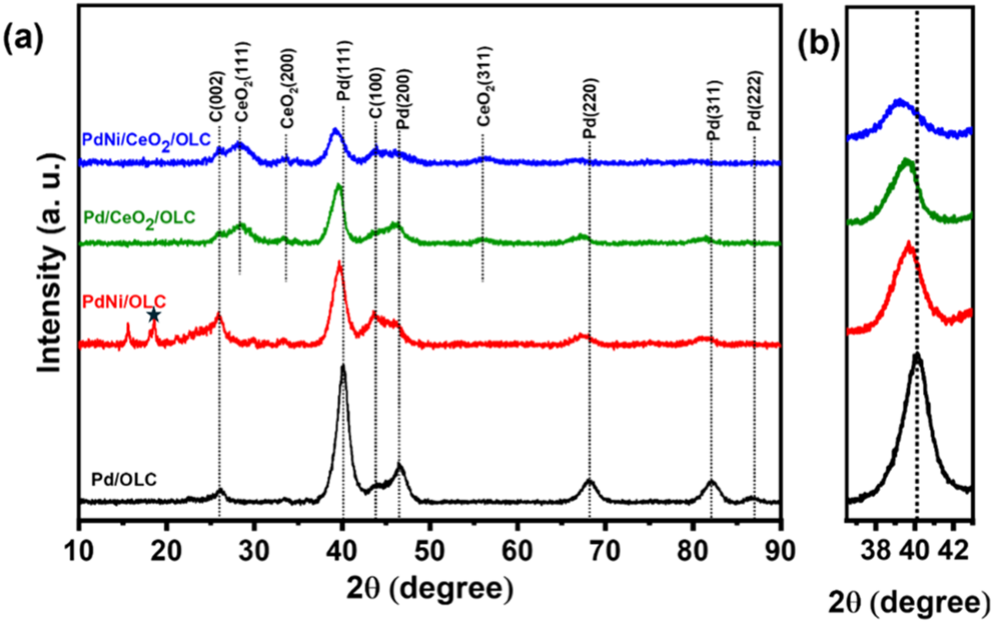
(a) Powder XRD peaks of Pd/OLC, PdNi/OLC, Pd/CeO_2_/OLC and PdNi/CeO_2_/OLC, and (b) the magnified plots of the peaks around 2θ angle of 40".

The diffraction patterns of the Pd/CeO_2_/OLC and PdNi/CeO_2_/OLC catalysts shifted to lower 2θ values of Pd(111) peak compared to Pd/OLC, suggesting lattice expansion in Pd due to the incorporation of CeO_2_, as indicated in Figure 2(b). This shift was attributed to the strong interfacial interactions between Pd and CeO_2_, which was further supported by the changes in the lattice constant of Pd. These structural modifications are correlated with the enhanced electrocatalytic performance. The diffraction patterns of the catalysts exhibit similar peak positions for the (111), (200), and (220) planes. However, a shift to lower 2θ values was observed for the PdNi/CeO_2_/OLC and Pd/CeO_2_/OLC catalysts compared to PdNi/OLC, indicating lattice expansion. The change in the crystal structure happens because CeO_2_ is much larger in size than Pd and Ni. When CeO_2_ is added, its bigger size causes a mismatch where it touches the smaller Pd and Ni particles. This size difference creates stress at the points where they connect, which pushes or pulls the atoms slightly out of place. As a result, the normal crystal structure of Pd and Ni gets changed. This change can be seen in XRD tests as a shift in the pattern. So, the large size of CeO_2_ causes strain that changes how the atoms are arranged in the material.

Table S2 compares the crystallite sizes and lattice constants of three different catalysts: PdNi/OLC, Pd/CeO_2_/OLC, and PdNi/CeO_2_/OLC. The crystallite sizes of the catalysts were calculated using the Scherrer equation, and the results show slight variations: PdNi/OLC has a crystallite size of 4.9 nm, Pd/CeO_2_/OLC measures 5.1 nm, and PdNi/CeO_2_/OLC is 4.7 nm. Regarding the lattice constants, a progressive increase was observed: PdNi/OLC has a lattice constant of 3.93 Å, Pd/CeO_2_/OLC of 3.95 Å, and PdNi/CeO_2_/OLC of 3.98 Å. This trend indicates that incorporating CeO_2_ into the catalyst slightly expanded the lattice structure.

Thermogravimetric analysis (TGA) and derivative thermogravimetry (DTG), shown in Figure S8, were used to study the thermal stability and metal content of the catalysts over a temperature range of 0–900 °C. A small weight loss below 150 °C is due to the evaporation of water that is weakly held on the surface. A larger weight loss happens around 600 °C, which is caused by the burning (oxidation) of the onion-like carbon (OLC) support. The total weight losses for PdNi/OLC, Pd/CeO_2_/OLC, and PdNi/CeO_2_ were about 75.8, 76.0, and 71.0%, respectively. The leftover mass (residue) after this step was approximately 24.2% for PdNi/OLC, 24.0% for Pd/CeO_2_/OLC, and 29.0% for PdNi/CeO_2_. These values represent the metals and metal oxides that remain.

The EDX analysis results shown in Figure S1 confirm the presence of elemental signals for Pd, Ni, CeO_2_, C, and O. Pd was added to all the catalysts at about 10 wt %. EDX analysis confirmed this, showing 9.43 wt % Pd in PdNi/OLC, 9.72 wt % in Pd/CeO_2_/OLC, and 9.19 wt % in PdNi/CeO_2_/OLC. In the PdNi/CeO_2_/OLC catalyst, Ni is also present at 10.92 wt %, showing that both metals were successfully added in nearly equal amounts. Although EDX gives semiquantitative results, it is still a useful tool for estimating composition. EDX mapping (Figures S2 and S3) also showed that Pd, Ni, and CeO_2_ were evenly spread throughout the catalyst, confirming that the synthesis method was effective. The DTG curves also help identify the temperatures at which major changes occur, providing additional information about the thermal behavior of the materials.

Figure 3 presents the HRTEM images of all the prepared nanostructure electrocatalysts, providing insights into their structures and morphologies. The low-magnification TEM images in Figure 4(a–c) reveal that the nanoparticles are well dispersed on OLC.

**Figure 3:**
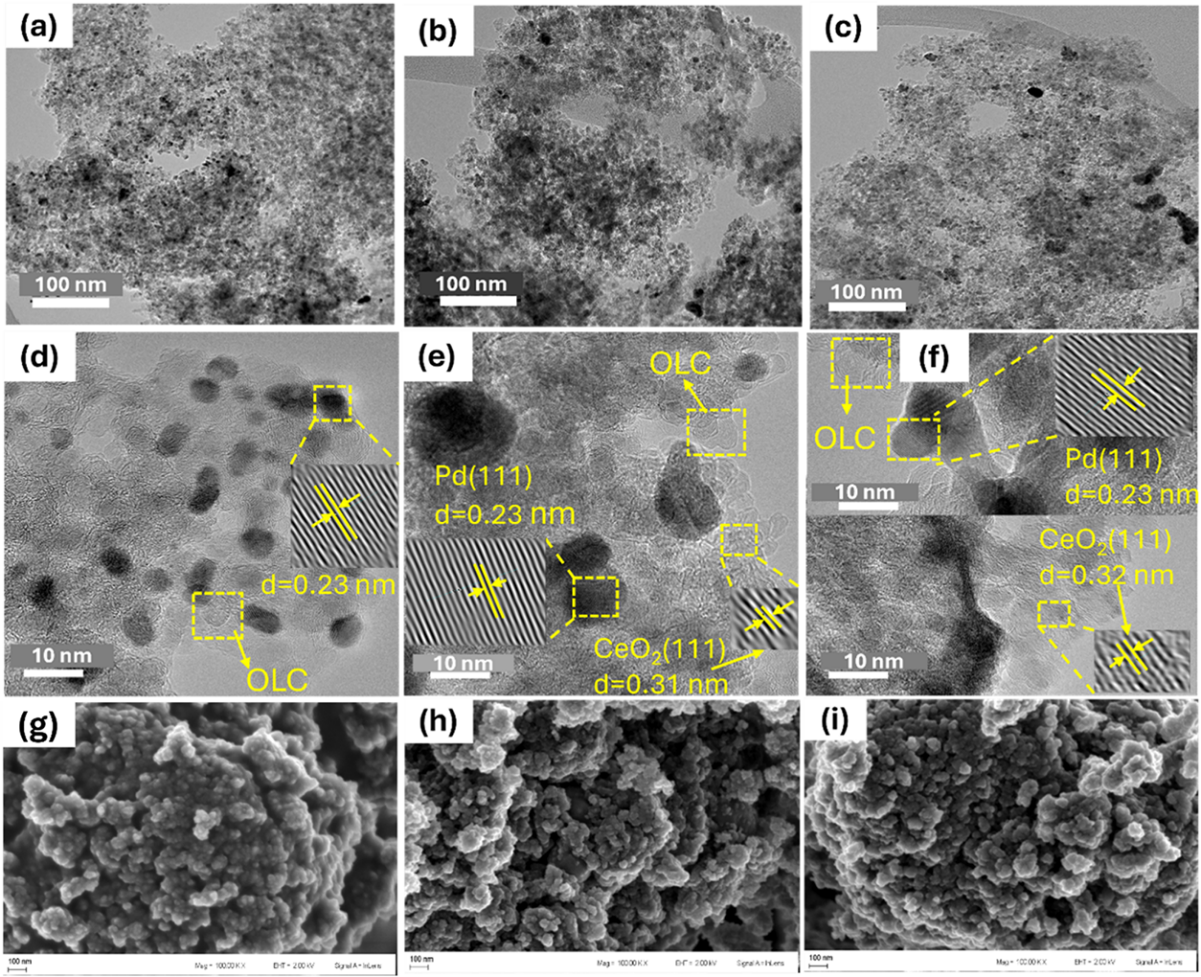
Low-magnification TEM images (a–c), high-resolution TEM (HRTEM) images (d–f), and SEM images (g–i) of the catalysts: (a, d, g) correspond to PdNi/OLC; (b, e, h) to Pd/CeO_2_/OLC; and (c, f, i) to PdNi/CeO_2_/OLC.

**Figure 4:**
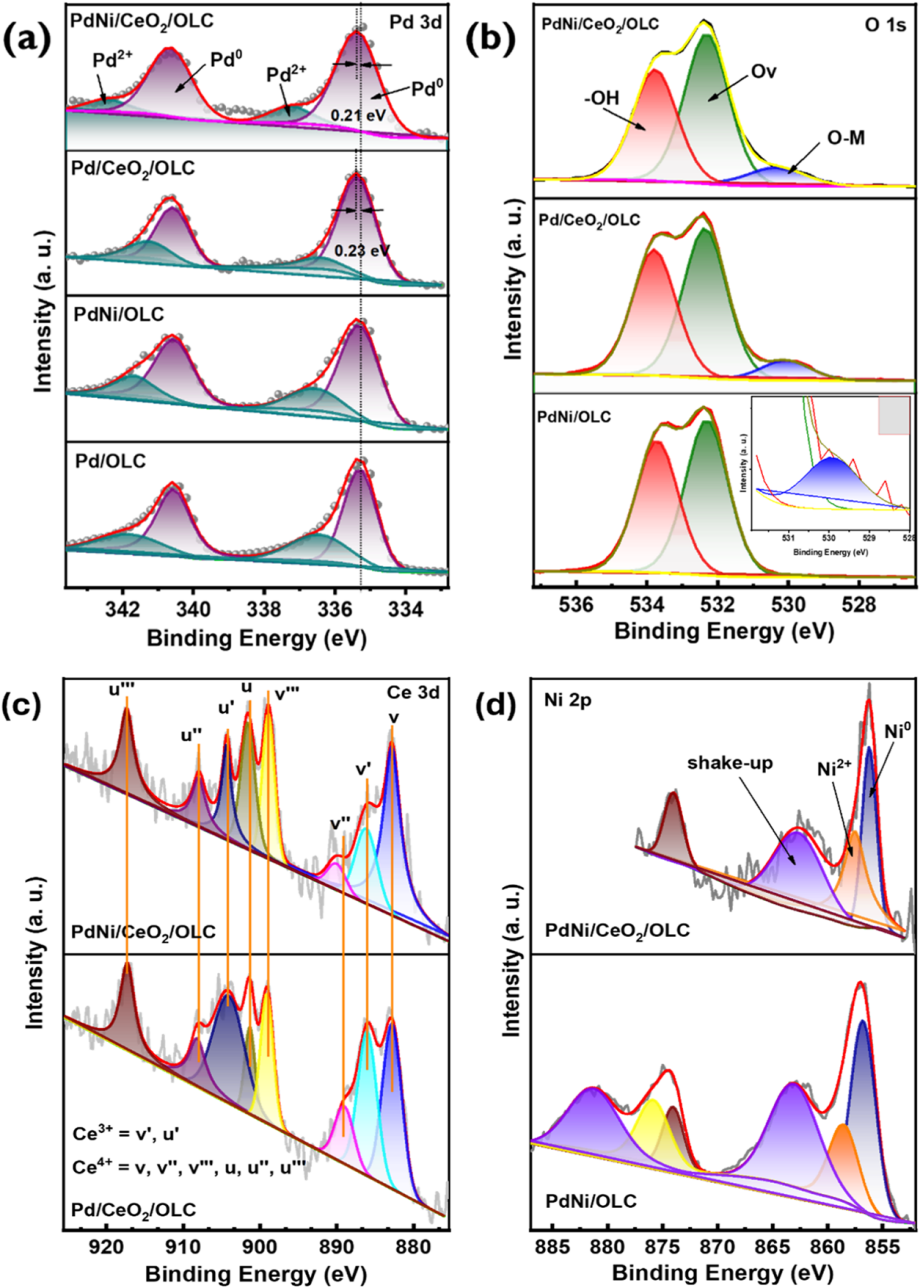
X-ray photoelectron spectra of the synthesized catalysts and Pd/C showing (a) the spectra of the Pd 3d energy peaks, (b) the O 1s spectra, (c) the Ce 3d spectra, and (d) the Ni 2p spectra energy peaks.

The primary support for the synthesized catalyst is onion-like carbons (OLC), which is characterized by a particle size in the nanometre range (<10 nm) and a spherical shape as shown in the HRTEM images in Figure 3(d–f). It is well established the OLC consist of multiple layers of graphitic carbon shells resembling fullerenes.^52,53^ Lattice distance was determined as 0.23 nm in all samples, indicating the presence of Pd(111), and 0.31 nm for CeO_2_ (111) in the Pd/CeO_2_/OLC and PdNi/CeO_2_/OLC catalysts, aligning with earlier findings.^54,55^ Additionally, SEM images in Figure 3(g–i) confirm that the synthesized catalysts have irregular shapes but exhibit consistent morphological features across the samples.

We also employed XPS to analyze the oxidation states of elements within the Pd 3d, O 1s, Ce 3d, and Ni 2p regions collected in Figure 4. The broad survey spectra collected within binding energies from 1400 to 0 eV confirm the presence of Pd, Ni, CeO_2_, O, and C in the catalysts as displayed in Figure S4­(a) and the C 1s spectra displayed in Figure S4­(b). To better understand how the addition of Ni and CeO_2_ affects the electronic properties of the active metal Pd, the Pd 3d spectra of the Pd/OLC, PdNi/OLC, Pd/CeO_2_/OLC, and PdNi/CeO_2_/OLC catalysts are shown in Figure 4(a). The spectra of all the catalysts reveal a doublet of Pd, which comprises a high-energy peak (3d_3/2_) and a low-energy peak (3d_5/2_). For Pd/C, Pd 3d_3/2_ and Pd 3d_5/2_ are at 335.08 and 340.46 eV, respectively. Both the Pd 3d_3/2_ and Pd 3d_5/2_ peaks shifted positively by 0.23 and 0.21 eV upon the incorporation of CeO_2_ into the Pd/C catalyst. This positive shift of Pd 3d_3/2_ and Pd 3d_5/2_ is due to the interactions with CeO_2_. Note that this electronic interaction may lead to d-band center shifting of the Pd and favor the IPA oxidation reaction activity. Significantly, the Pd^0^/Pd^2+^ spectra for PdCeO_2_/OLC shifted to lower binding energies than those of Pd/C and PdNi/OLC. This shift is attributed to the withdrawal of electrons of Pd by CeO_2_, which reduces the electron density but enhances the interfacial interactions.

The O 1s XPS peak in Figure 4(b) at 530.4 eV corresponds to oxygen in metal–oxygen (M-O) bonds, which is characteristic of the O^2–^ ions in the surface lattice of the CeO_2_ matrix, as well as in Pd and Ni oxides. The higher binding energy peak around 532.2 eV (Ov) is attributed to oxygen defects, specifically oxygen vacancies, in the metal oxide matrix. These anionic vacancies alter the overall electronic charge density. This nonlattice oxygen peak has been linked to surface O^–^ ions with a lower electron density. The peak at 533.7 eV is associated with the surface hydroxyl groups.^56–58^

The Ce 3d spectrum for Pd/CeO_2_/OLC and PdNi/CeO_2_/OLC in Figure 4(c) displays several peaks, indicating the splitting of electronic states into Ce 3d_5/2_ and Ce 3d_3/2_. It also reveals the different oxidation states of CeO_2_, specifically Ce^4+^ and Ce^3+^. The positions and attributions of the lines are as follows: 3d_5/2_: u, u′, and u‴, and 3d_3/2_: v, v″, and v‴ for Ce^4+^ cations, and 3d_5/2_: u′ and 3d_3/2_: v′ for Ce^3+^ cations.^59^ Analysis of the Ni 2p XPS peaks in the catalyst showed two peaks: one at 856 eV for Ni^0^ 2p_3/2_, one at 859 eV for Ni^2+^ 2p_3/2_ (Figure 4(d)).^60^

In CeO_2_-based catalysts, the O 1s peak shifts to lower binding energies with broadened peak. Specifically, the binding energy shifts by 0.91 and 0.67 eV for Pd/CeO_2_/OLC and PdNi/CeO_2_/OLC, respectively. Lattice oxygen is small in intensity for PdNi/OLC electrocatalyst.

### Electrochemical Characterization

#### Bare Electrode Response in KOH

To probe the redox properties of the catalysts, cyclic voltammetry (CV) was performed in a nitrogen-saturated 0.5 M KOH electrolyte solution, using a scan rate of 50 mV/s over 20 cycles. The resulting CV curves, recorded within a potential range of −1.00 to 0.40 V vs Ag/AgCl at ambient temperature, are presented in Figure 5(a). In the forward scan, a broad, small peak in the lower potential region was observed, corresponding to hydrogen desorption and hydroxyl (HO) adsorption on the catalyst surface. A peak indicative of PdO formation appeared in the higher potential range (−0.10 to 0.20 V). During the reverse scan, distinct peaks at −0.20 to −0.50 V and −0.60 to −0.90 V vs Ag/AgCl are attributed to the reduction of PdO and hydrogen adsorption (H_ad_), respectively. Additionally, peaks observed above +0.35 V are associated with the redox processes involving OH^–^ adsorption and Ni oxide during both anodic and cathodic scans. Specifically, a current density increase of +0.35 V during the forward scan reflects the oxidation of Ni^2+^ in Ni­(OH)_2_ to Ni^3+^ in NiOOH. In the reverse scan, Ni^3+^ was reduced to Ni^2+^ in the nickel-containing catalysts. These findings suggest a reversible redox reaction, represented by the following equation

$$\mathrm{Ni}{\left(\mathrm{OH}\right)}_{2}+{\mathrm{OH}}^{-}⇌\mathrm{NiOOH}+H_{2}O+e^{-}$$

**Figure 5:**
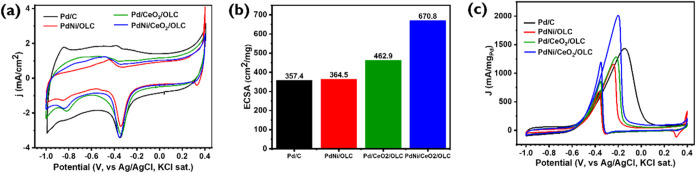
CVs of catalysts in (a) 0.5 M KOH solutions and (b) their corresponding ECSA values, and (c) cyclic voltammograms obtained in 0.5 M KOH + 1.0 M IPA solutions using different GCE-modified catalysts.

The anodic scans, revealing that all the synthesized catalysts exhibit a negative shift in peak potentials relative to Pd/C and in the cathodic scans, both PdNi/OLC and PdNi/CeO_2_/OLC catalysts exhibited a more positive shift in their cathodic peak potentials. In contrast, the Pd/CeO_2_/OLC catalyst maintained peak potentials equivalent to that of the Pd/C catalyst. The reduction peak of PdO observed in the potential range of −0.50 to −0.20 V vs Ag/AgCl is related to the electrochemical active surface area (ECSA)_Pd_ of the catalyst electrode. ECSA is a key parameter for assessing catalyst performance, as a larger ECSA signifies a greater active surface area for the oxidation of IPA.

ECSA was determined using the following equation^54^

3 $$\mathrm{ECSA}=\frac{Q_{\mathrm{Pd}}}{\mathrm{SL}}$$

where *Q* is the Coulombic charge of the Palladium reduction peak, was obtained by integrating the area under each peak, *S* is the Coulombic constant for a monolayer of Pd (0.424 mC cm), and *L* is the loading of the metal catalysts on surface of the electrode (2 μg of Pd).

Figure 5(b) shows the calculated ECSA values for the synthesized electrocatalysts and commercial Pd/C. The ECSA values are as follows: 357.4 cm^2^/mg for Pd/C, 364.5 cm^2^/mg for PdNi/OLC, 462.9 cm^2^/mg for Pd/CeO_2_/OLC, and 670.8 cm^2^/mg for PdNi/CeO_2_/OLC which is calculated using eq 3. A higher ECSA reflects greater dispersion of Pd nanoparticles, suggesting enhanced catalytic activity. Notably, the PdNi/CeO_2_/OLC catalyst exhibits the highest ECSA, as evidenced by the significant charge associated with the PdO reduction peak. This indicates that the Pd nanoparticles on the CeO_2_ support are highly electrochemically active, with PdNi/CeO_2_/OLC demonstrating the highest activity among the tested catalysts.

#### Electrocatalytic Activity toward IPA Oxidation

The catalytic activities of the catalysts for IPA oxidation were evaluated using CV measurements in a 0.5 M KOH + 1.0 M IPA solution at a scan rate of 50 mV/s over a potential range of −1.00 to 0.40 V. The CV profiles of the synthesized electrocatalysts and the commercial Pd/C catalysts (see Figure 5(c)) demonstrate distinct forward (*j*_f_) and backward (*j*_b_) currents, where the forward current reflects the electroactivity for IPA oxidation and was observed as a peak at approximately −0.20 V (vs Ag/AgCl sat. KCl), and the backward current indicates the oxidation of poisonous intermediates, evidenced by a peak around −0.35 V (vs Ag/AgCl, 3 M KCl). The electrochemical parameters obtained from the measurements of the IPA oxidation process are summarized in Figure 5(c) and Table 1.

**Table 1:** Electrochemical Performance of IPA Oxidation on the Synthesized Catalysts Measured in 1.0 M IPA and 0.5 M KOH Solution at a Scan Rate of 50 mV/s

catalyst	*E*_onset_ (V)	Δ*E* (V)	*j*_f_ (mA/mg_Pd_)	Tafel slope (mV/dec)
Pd/C	–0.64	–0.212	1495.9	200.2
PdNi/OLC	–0.68	–0.126	1150.6	121.5
Pd/CeO_2_/OLC	–0.70	–0.137	1281.8	204.5
PdNi/CeO_2_/OLC	–0.69	–0.149	2017.3	136.9

Analysis of the alcohol oxidation performance of the various catalysts revealed significant differences in their electrochemical characteristics.^61^ As shown in Table 1, Pd/C exhibited a peak separation of −0.212 V, indicating slower electron transfer kinetics despite a relatively high forward peak intensity of 1495.9 mA/mg_Pd_, which reflects its ability to facilitate the oxidation reaction. In contrast, PdNi/OLC demonstrated markedly improved peak separation with −0.126 V, suggesting enhanced kinetics for electron transfer, although its forward peak intensity of 1150.6 mA/mg_Pd_. Pd/CeO_2_/OLC provided intermediate performance with a peak separation of −0.137 V and an intensity of 1281.8 mA/mg_Pd_, reflecting improved kinetics over Pd/C while still maintaining effective catalytic activity. Notably, PdNi/CeO_2_/OLC exhibited a peak separation of −0.149 V and the highest forward peak intensity in 2017.3 mA/mg_Pd_, indicating both efficient electron transfer and superior oxidation capabilities. This indicates that the combination of nickel with ceria in Pd-based catalysts significantly enhances the overall IPA oxidation performance, optimizing both kinetics and reaction rates. Table 2 presents a comparison between the synthesized electrocatalyst for IPA electrooxidation and those previously reported in the literature.

**Table 2:** Comparison of Pd-Based Electrocatalysts for IPA Electrooxidation

	material	*E*_s_ (V)	*E*_p_ (V)	*J*_f_ (mA/mg)	refs
1	Pd-CNT	–0.608 vs Ag/AgCl	–0.300 vs Ag/AgCl	366.6 mA/mg	^28^
2	Pd-CB	–0.626 vs Ag/AgCl	–0.286 vs Ag/AgCl	406.9 mA/mg	^28^
3	Pd-1CNT-Ni-1CB	–0.646 vs Ag/AgCl	–0.285 vs Ag/AgCl	795.0 mA/mg	^28^
4	Pd-1CNT-Ni-2CB	–0.643 vs Ag/AgCl	–0.270 vs Ag/AgCl	1204.9 mA/mg	^28^
5	Pd-2CNT-Ni-1-CB	–0.626 vs Ag/AgCl	–0.290 vs Ag/AgCl	532.8 mA/mg	^28^
6	Pd-1CNT-2CB	–0.596 vs Ag/AgCl	–0.271 vs Ag/AgCl	500.2 mA/mg	^28^
7	Pd–Nb/CNTs	0.50 vs RHE	NA	776 mA/mg	^31^
8	Pd/CNTs	0.50 vs RHE	NA	470 mA/mg	^31^
9	Pd/C	0.60 vs RHE	NA	267 mA/mg	^31^
11	PdAu41/C	–0.68 vs Ag/AgCl	–0.32 vs Ag/AgCl	NA	^62^
12	PdAu21/C	–0.69 vs Ag/AgCl	–0.31 vs Ag/AgCl	NA	^62^
13	PdAu21/C	–0.68 vs Ag/AgCl	–0.31 vs Ag/AgCl	NA	^62^
14	Pd electrode	–0.78 vs SCE	–0.39 vs SCE	52 mA/cm^2^	^30^
15	Pt electrode	–0.72 vs SCE	–0.30 vs SCE	4 mA/cm^2^	^30^
16	Pd at 50 mV/s	–0.68 vs SCE	–0.323 vs SCE	103 mA/cm^2^	^63^
17	Pd (bulk)	NA	–0.4 vs RHE	52 mA/cm^2^	^64^
18	Pt (bulk)	NA	–0.4 vs RHE	4 mA/cm^2^	^64^
19	Pd–Ni foam	NA	–0.4 vs RHE	64 mA/cm^2^	^65^
20	Pd/C	–0.64 vs Ag/AgCl	–0.15 vs Ag/AgCl	1496.9 mA/mg	This Work
21	PdNi/OLC	–0.68 vs Ag/AgCl	–0.24 vs Ag/AgCl	1150.6 mA/mg	This work
22	Pd/CeO_2_/OLC	–0.70 vs Ag/AgCl	–0.22 vs Ag/AgCl	1281.8 mA/mg	This work
23	PdNi/CeO_2_/OLC	–0.69 vs Ag/AgCl	–0.20 vs Ag/AgCl	2017.3 mA/mg	This work

All synthesized catalysts display a lower onset potential compared to commercial Pd/C, which is −0.64 V. This suggests that the synthesized catalysts require less energy for electrooxidation of IPA. Notably, the PdNi/CeO_2_/OLC catalyst demonstrated the highest current response among the tested catalysts, indicating its superior kinetics for IPA oxidation. Previous research highlights the significance of a low onset potential for fuel cell applications, as it leads to a reduced anode potential, ultimately resulting in a higher open circuit potential for direct IPA fuel cell, DIFC.^15,62^

Figure S6, illustrates the scan rate study of the electrocatalysts starting from 20 mV/s to 200 mV/s with the interval of 20 mV/s. Figure 6(a) illustrates the relationship between the peak current of IPA oxidation on the electrocatalysts and the square root of the sweep rate. A linear correlation indicating that the oxidation process is governed by diffusion. This relationship between *i*_p_ and *v* ^1/2^ is described by the Randles–Ševčík equation, eq 4.

4 $$i_{p}=2.69\times 10^{5}n^{3/2}\mathrm{AC}D^{1/2}\upsilon^{1/2}$$

where *D* ^1/2^ is the diffusion coefficient in cm/s^2^, *A* is the electrode surface area in cm^2^, *C* is the concentration in m mol/L, *v* is the sweep rate in mV/s, and n the total number of electrons involved in the electrode process.

**Figure 6:**
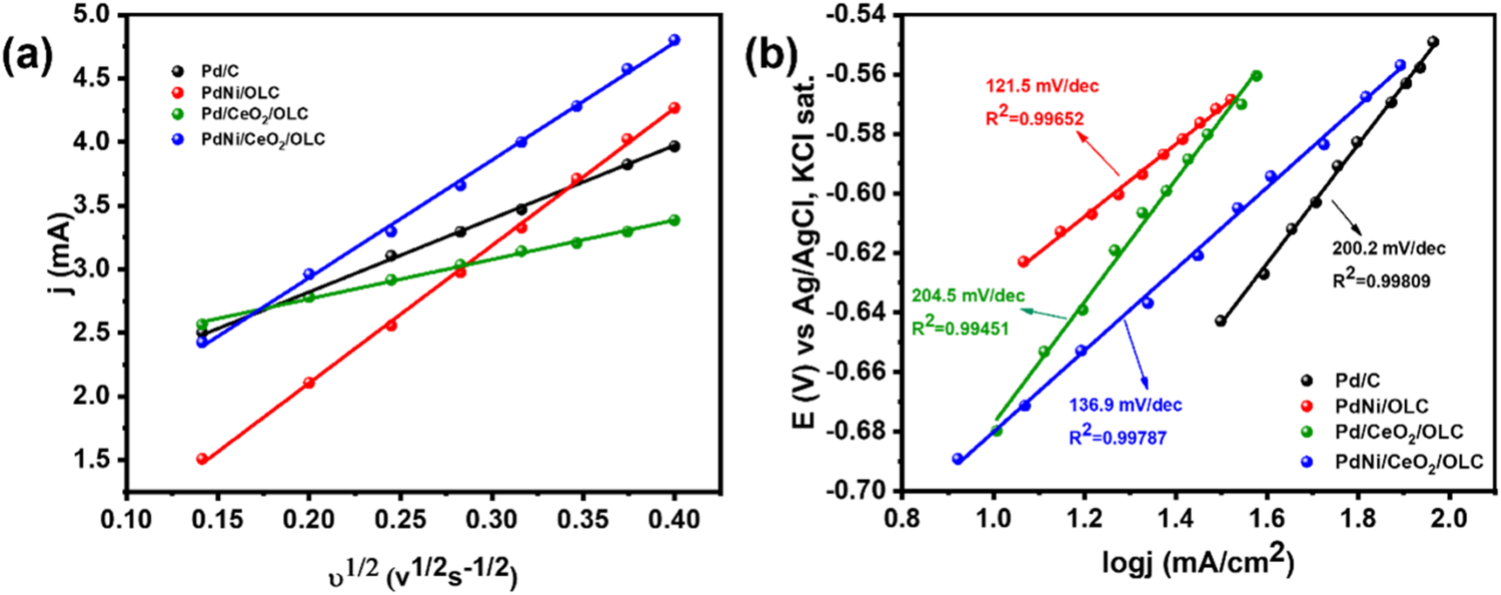
(a) Relationship of peak current of IPA oxidation on the synthesized catalysts and square root of sweep rate and (b) Tafel plot.

The slopes derived from the Randles–Ševčík Equation in the cyclic voltammetric analysis of IPA oxidation highlight the significant role of Ni in enhancing the performance of electrocatalysts. The slopes for PdNi/OLC (10.80) and PdNi/CeO_2_/OLC (9.24) were relatively similar, demonstrating that the presence of nickel in these catalysts contributed to the effective diffusion of IPA to the electrode surface. In contrast, the slopes for Pd/CeO_2_ (3.09) and commercial Pd/C (5.76) are notably lower, indicating that these materials are less efficient in facilitating mass transport. The superior performance of the PdNi-based catalysts underscores the importance of Ni in promoting enhanced catalytic activity and diffusion properties during IPA oxidation. Ni may improve the electronic properties and active surface area of the catalyst, thereby facilitating better mass transport. The comparable slopes for PdNi/OLC and PdNi/CeO_2_/OLC suggest that the addition of cerium oxide does not compromise the beneficial effects of nickel. Overall, these findings emphasize that incorporating Ni into the catalyst design is crucial for optimizing the electrochemical performance, with PdNi-based catalysts demonstrating significantly better diffusion efficiency for IPA oxidation under the same concentration conditions (0.5 M KOH and 1.0 M IPA).

#### Tafel Plot

To compare the kinetic activities of the Pd/C, PdNi/OLC, Pd/CeO_2_/OLC, and PdNi/CeO_2_/OLC catalysts in the IPA oxidation reaction, we use Tafel slope. Figure 6(b) illustrates the relation between the potential vs the logarithmic current density (Tafel plots) for these synthesized catalysts alongside commercial Pd/C in a reaction medium of 0.5 M KOH and 1.0 M IPA. Tafel slopes were determined using linear sweep voltammetry (LSV) at a scanning rate of 10 mV/s. The linear region of the Tafel plots stretches from −0.70 to −0.54 V. As the potential is further increased above −0.54 V, the Tafel plot becomes curved, indicating the IPA oxidation reactions are no longer charge-transfer-controlled reactions. Tafel slopes derived from the linear region are summarized in Table 1. The results show Tafel slopes of 200.2 mV/dec for Pd/C, 121.5 mV/dec for PdNi/OLC, 204.5 mV/dec for Pd/CeO_2_/OLC, and 136.9 mV/dec for PdNi/CeO_2_/OLC. A lower Tafel slope value indicates faster charge-transfer kinetics,^66^ highlighting the superior catalytic performance of PdNi/OLC and PdNi/CeO_2_/OLC for facilitating the IPA oxidation reaction. Moreover, the decreases in Tafel slope might be due to the presence of Ni on the electrocatalysts.

#### Electrochemical Impedance Spectroscopy (EIS)

EIS is an effective technique for investigating the electrocatalytic properties of materials, particularly for assessing ion transport, diffusion, and electron kinetics at the electrode/electrolyte interface in fuel cells.^67–69^ EIS enables the study of electron transfer mechanisms and aids in the understanding of the electrochemical processes involved in catalysis. Nyquist plots were generated and analyzed to elucidate the mechanism underlying the enhanced IPA oxidation. EIS measurements were conducted in the frequency range of 200 kHz to 100 mHz in a solution of 0.5 M KOH and 1.0 M IPA. Nyquist plots are commonly used to evaluate the charge-transfer resistance (R_ct_), which can be extracted from the diameter of the semicircular arc, providing insight into the reaction kinetics. Figure 7(a) presents the EIS spectra of the various catalysts in 0.5 M KOH and 1.0 M IPA solutions. The Nyquist plots of the prepared catalysts exhibited a typical semicircular shape, and the electrical circuit model used to fit the data included the solution resistance (*R*_s_), charge transfer resistance (*R*_ct_), and constant phase element (CPE), which represent the characteristics of the electrolyte-electrode interface during the IPA oxidation reaction. The arc radius in EIS is directly related to the charge transfer resistance (*R*_ct_) between the electrode and the electrolyte. A smaller arc radius indicates a reduced electron transfer resistance, implying more efficient electrocatalysis.

**Figure 7:**
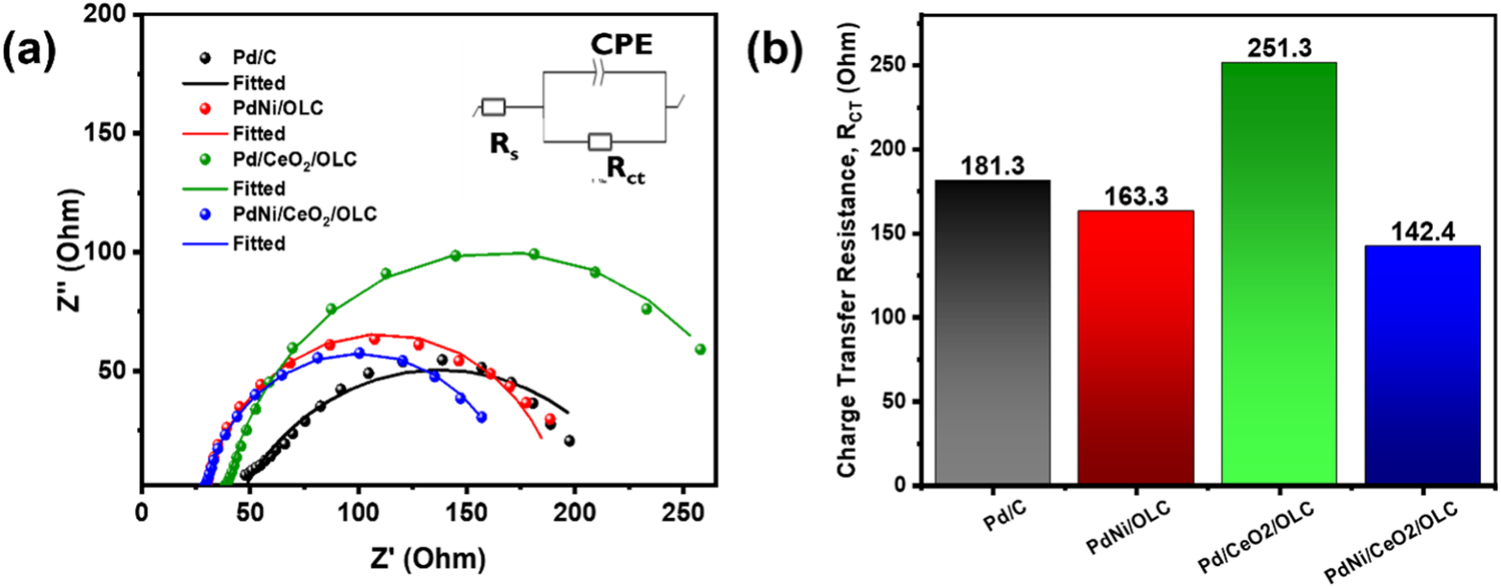
(a) Potentiostatics EIS pattern of IPA oxidation on the catalysts at a potential of −0.3 V and (b) Charge Transfer Resistance (R_ct_) value of IPA oxidation.

The Pd/CeO_2_/OLC catalyst exhibited the highest charge-transfer resistance (*R*_ct_) of 251 Ω, indicating sluggish kinetics for IPA oxidation. In contrast, the PdNi/CeO_2_/OLC catalyst exhibited the lowest *R*_ct_ value (142.4 Ω) suggesting faster electron transfer and more favorable oxidation kinetics. The smaller *R*_ct_ value reflects faster electron transfer, suggesting that PdNi/CeO_2_/OLC has superior electron conductivity and ion transport compared to other electrocatalysts. These findings suggest that PdNi/CeO_2_/OLC demonstrates superior electrocatalytic performance, as confirmed by its smaller arc diameter and lower solution resistance, which are consistent with the enhanced ECSA, CV, and CA results. The synergistic effect between Ni and CeO_2_ in the PdNi/CeO_2_/OLC catalyst is likely responsible for the observed improvement in IPA oxidation activity.

The equivalent electrical circuit includes the solution resistance (*R*_s_), charge-transfer resistance (*R*_ct_), and constant phase element (CPE). The fitting curves, shown in Figure 7(a), demonstrate a good match between the experimental data and the equivalent circuit model, confirming the validity of the analysis. Figure 7(b) shows the *R*_ct_ values for IPA oxidation on Pd/C, PdNi/OLC, Pd/CeO_2_/OLC, and PdNi/CeO_2_/OLC catalysts are 181.3 Ω, 163.3 Ω, 251.3 Ω, and 142.4 Ω, respectively. These results indicate that the PdNi/CeO_2_/OLC catalyst exhibited significantly enhanced electroactivity for IPA oxidation compared to the other catalysts.

#### Chronoamperometry

The long-term reactivity of IPA on Pd/C, PdNi/OLC, Pd/CeO_2_/OLC, and PdNi/CeO_2_/OLC catalysts was investigated using chronoamperometry (CA) in a 0.5 M KOH and 1.0 M IPA solution at a constant potential of −0.3 V (vs Ag/AgCl, KCl sat.) for 3600 s, to assess the stability of the electrocatalysts.^69,70^ The chronoamperograms in Figure 8(a), were used to assess resistance to poisoning during the oxidation of IPA on the catalyst surfaces.

**Figure 8:**
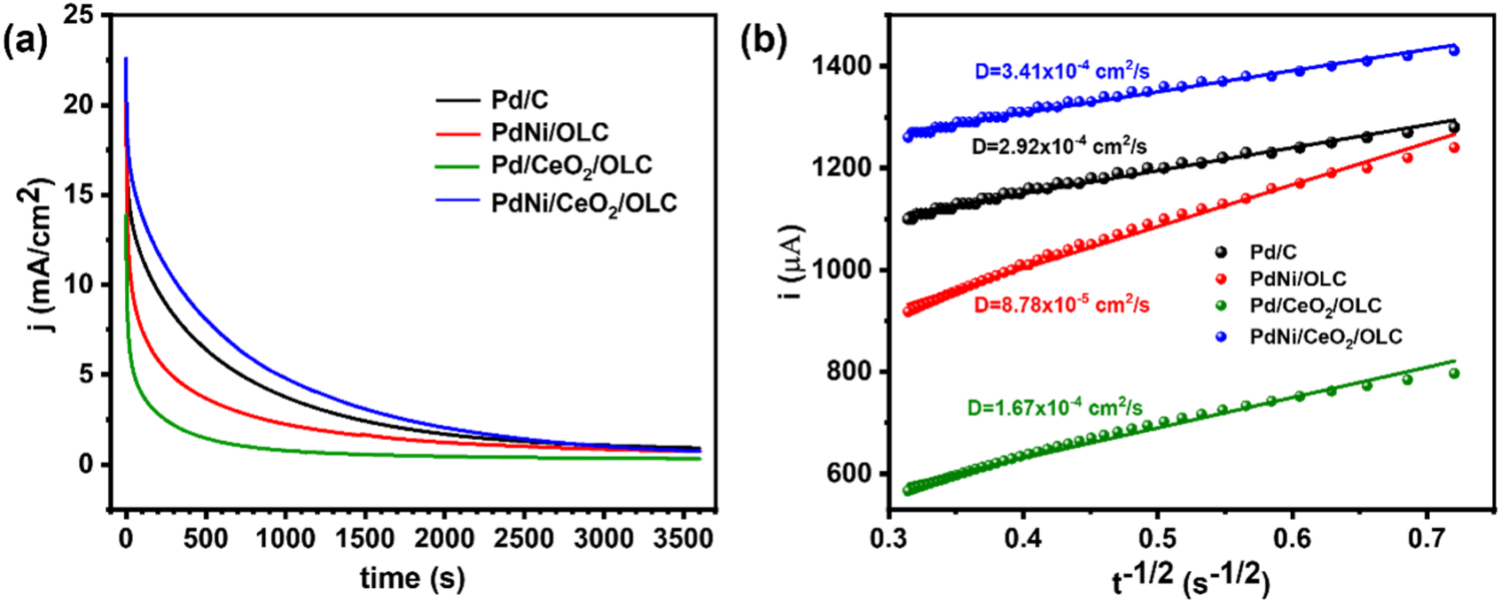
(a) Chronoamprograms of the synthesized catalysts and (b) Cottrell plot (*i* vs *t* ^–1/2^) of the chronoamperometric data obtained for the synthesized electrocatalysts.

The polarization current of Pd/CeO_2_/OLC decreased rapidly within the first 100 s, which can be attributed to the accumulation of strongly adsorbed reaction intermediates on the surface-active sites. It then gradually decayed until it reached the limiting current at 3600 s. In contrast, PdNi/OLC exhibited a decline over 1000 s, followed by Pd/C, showing a similar trend. Notably, PdNi/CeO_2_/OLC remained stable for a much longer duration than the other catalysts, demonstrating superior durability.

From Figure 8(a), we can conclude that the order of activity for IPA oxidation in terms of current density is PdNi/CeO_2_/OLC > Pd/C > PdNi/OLC > Pd/CeO_2_/OLC. This analysis indicates that the PdNi/CeO_2_/OLC catalyst is generally more stable than the other electrocatalysts, highlighting the significance of Ni and CeO_2_ with OLC in enhancing the catalyst stability. Additionally, the combination of OLC with Ni and CeO_2_ improved the antipoisoning capability and catalyst durability, which aligns with previous study on the oxidation of alcohols.^54^

To calculate the diffusion coefficient from CA, we use Cottrell equation.

5 $$i=\frac{\mathrm{nFAC}}{\sqrt{\pi \mathrm{Dt}}}$$

where *i* is the current, *n* is the number of electrons involved in the electrochemical reaction which is 2 for IPA oxidation to acetone, *F* is Faraday’s constant (96485 C/mol), *A* is the electrode area (0.0707 cm^2^), the concentration of IPA is 0.5 mol/L = 1 × 10^–3^ mol/cm^3^, *D* is the diffusion coefficient and t is the time in seconds.^71^

The diffusion coefficients for Pd/C, PdNi/OLC, PdCeO_2_/OLC, and PdNi/CeO_2_/OLC were calculated as 7.30 × 10^–5^, 2.20 × 10^–5^, 4.18 × 10^–5^ and 8.53 × 10^–5^ cm^2^/s, respectively. As shown in Figure 8(b), the PdNi/CeO_2_/OLC electrocatalyst exhibited the highest diffusion coefficient among the tested materials. This suggests that PdNi/CeO_2_/OLC allows for faster ion or molecule diffusion than Pd/C, PdNi/OLC, and Pd/CeO_2_/OLC. The enhanced diffusion in PdNi/CeO_2_/OLC indicated that the combination of PdNi and CeO_2_ improved the ability of the material to transport ions or molecules, potentially enhancing its performance in applications such as catalysis and energy storage. In addition, this result suggests that impurities or products can be more easily desorbed from the surface of the PdNi/CeO_2_/OLC electrocatalyst because of faster diffusion, which can improve its long-term stability and efficiency. In contrast, PdNi/OLC and PdCeO_2_/OLC had lower diffusion coefficients, reflecting slower diffusion rates for the oxidation of IPA.

#### DFT Calculations

Figure 9 illustrates the configuration of IPA adsorbed on four electrocatalyst models. Figures 9(a,b) display the Pd(111) and PdNi(111) surfaces, modeled using a (4 × 4) primitive supercell with four slab layers. In the PdNi(111) model, Pd and Ni atoms are uniformly distributed across all four layers in a 1:1 ratio, aligning with experimental synthesis.

**Figure 9:**
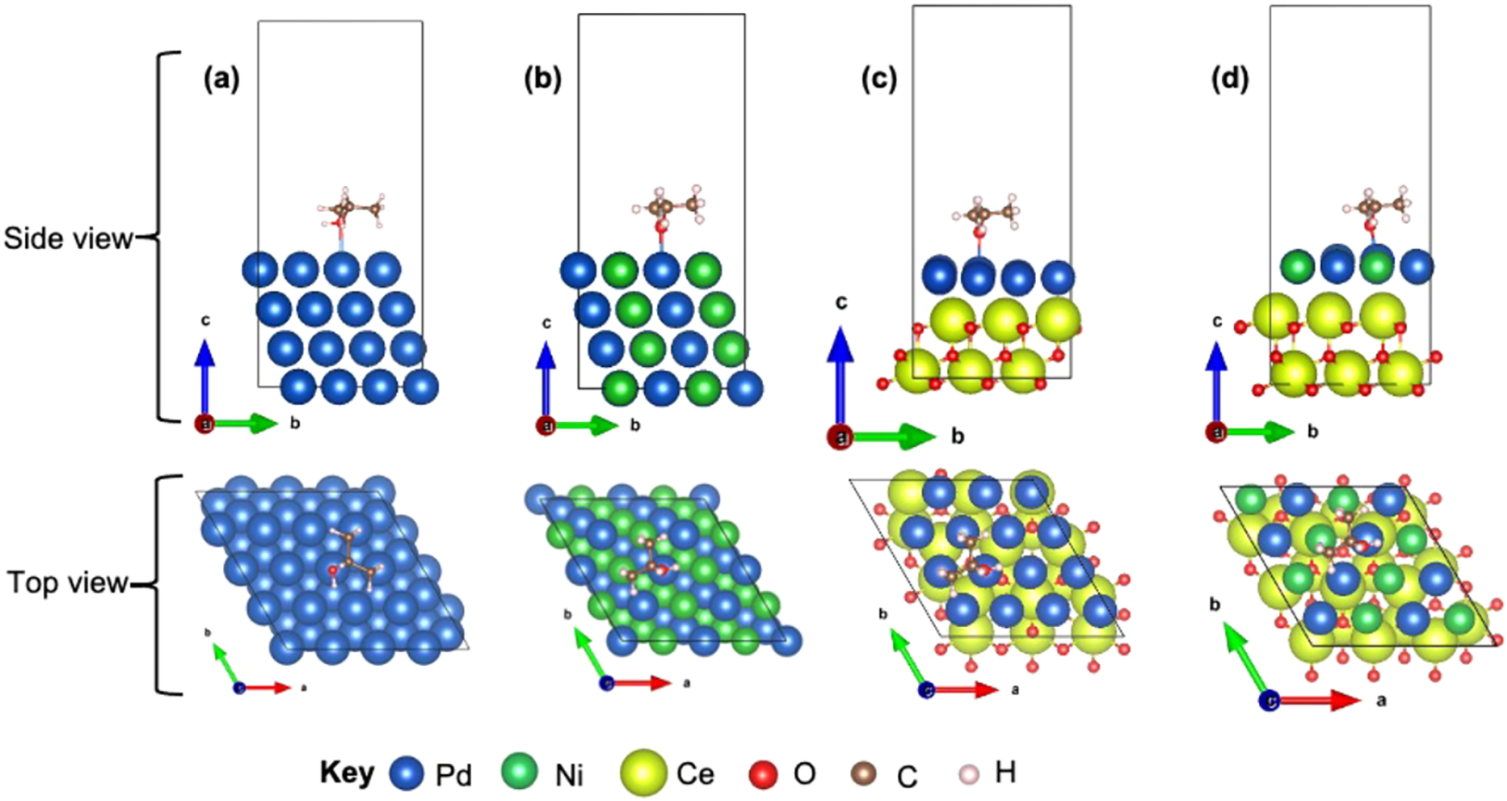
Optimized structure of the (a) Pd(111), (b) PdNi(111), (c) Pd(111)/CeO_2_, and (d) PdNi(111)/CeO_2_ structures.

Figure 9 (c,d) depict the heterostructures Pd/CeO_2_ and PdNi/CeO_2_, respectively. To construct these models, a 4:3 lattice-matching scheme was employed, where a (4 × 4) primitive single layer of Pd(111) or PdNi(111) was placed on a two-layer (3 × 3) primitive CeO_2_(111) substrate, forming the respective heterostructures. In the PdNi/CeO_2_ model, Pd and Ni atoms maintain a 1:1 distribution within the topmost layer, again reflecting experimental synthesis.

The bulk lattice constants for Pd and CeO_2_ are 3.99 and 5.48 Å, respectively, consistent with previous studies.^72,73^ A CeO_2_(3 × 3) primitive supercell was constructed to achieve a 1.66% lattice mismatch with the (4 × 4) primitive Pd(111) and PdNi(111) surfaces. This lattice alignment is consistent with their face-centered cubic (FCC) crystal structures, as supported by both theoretical calculations and experimental data.^74,75^

The distances between the oxygen atom of IPA and the top Pd atom on the surface of the electrocatalysts are shown in Table 3. The values are 2.41, 2.64, 2.35, and 2.38 Å for Pd(111), PdNi(111), Pd(111)/CeO_2_, and PdNi(111)/CeO_2_, respectively. These values are closer to those reported in an earlier study on the Pd(111) surface with 2.21 Å.^76^

**Table 3:** Adsorption Energies Calculated for the IPA-Pd Configuration and Minimum Distance (d) between the O Atom on the IPA and Pd on the Surface

configurations	*E*_ad_ (eV)	*d* (Å)	d-band center (eV)
IPA-Pd(111)	–1.51	2.41	–2.73
IPA-PdNi(111)	–1.23	2.64	–2.59
IPA-Pd(111)/CeO_2_	–2.08	2.35	–3.22
IPA-PdNi(111)/CeO_2_	–1.82	2.38	–2.49

The electronic properties and adsorption characteristics of the Pd(111) and PdNi(111) alloys were systematically studied, focusing on their interactions with CeO_2_ as a support. Figure 10 (a–d) shows the projected density of states (PDOS) for the interaction between oxygen (O 2p orbital) of the IPA and palladium (Pd 4d orbital) on the (111) surface of each catalyst with the Fermi level (*E*_f_) set at 0.0 eV. There is a significant overlap in the energy levels from approximately −6.0 to 1.0 eV for both Pd(111) and PdNi(111), and from −6.0 to 2.0 eV for Pd(111)/CeO_2_ and PdNi(111)/CeO_2_. This overlap, along with the weak antibonding interactions observed from 0 to 2 eV, suggests a strong bonding interaction between IPA and Pd sites on the (111) surface of each catalyst. The d-band centers were calculated using eq 2 for pure Pd(111) and PdNi(111), giving values of −2.73 and −2.59 eV, respectively (see Figure 10(a,b)). The lower d-band center in PdNi(111) (−2.59 eV) indicates a greater availability of d-states for bonding, enhancing its reactivity. When supported on CeO_2_, the d-band centers shift from −3.22 eV for Pd (away from the Fermi level) to −2.49 eV for PdNi toward the Fermi level (see Figure 10(c,d)). This shift highlights the significant impact of the ceria support on the electronic environment, optimizing the reactivity of the PdNi alloy. The lower d-band center of Pd/CeO_2_ suggests reduced electron availability. In contrast, the PdNi/CeO_2_ system retains a relatively higher d-band center compared to all of them, improving its electron donation capability owing to the presence of Ni.

**Figure 10:**
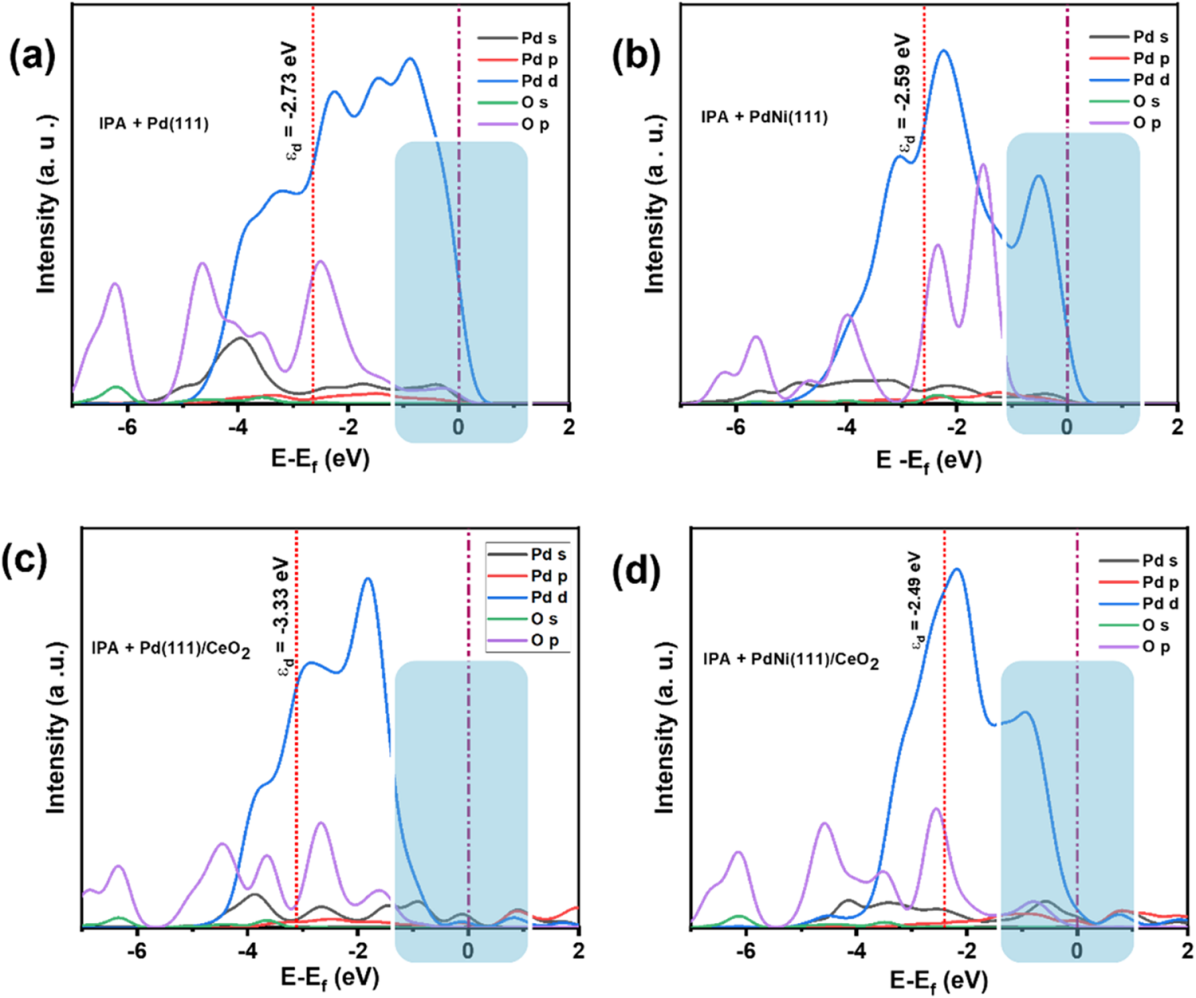
PDOS analysis for IPA on (111) surfaces of the (a)­Pd(111), (b)­PdNi(111), (c)­Pd(111)/CeO_2_, and (d)­PdNi/CeO_2_/OLC.

The PDOS at the Fermi level is crucial for the adsorption of IPA and significantly affects the electrocatalytic activity of the catalysts. The highlighted regions in Figure 10 also demonstrates that the availability of the d-orbital of Pd near the Fermi level for the electrocatalysts which is relatively low in the Pd/CeO_2_ electrocatalyst. This observation is supported by the XPS results, which show a shift of Pd^0^ to a higher binding energy in the presence of CeO_2_ as a support, with a value of approximately 0.2 eV. This suggests that CeO_2_ draws electrons from the Pd surface. In contrast, when Ni was present in the PdNi and PdNi/CeO_2_ electrocatalysts, the intensity of the Pd d-orbital near the Fermi level was significantly higher. This increase was attributed to the electron donation from Ni to Pd. The presence of Ni enhanced the electron density around Pd, leading to a higher availability of Pd d-orbitals, which could positively affect the catalytic activity of the PdNi and PdNi/CeO_2_ systems.

In addition, CeO_2_ containing catalysts (Pd/CeO_2_ and PdNi/CeO_2_) exhibit more available orbitals in the conduction band, which further contributes to their enhanced catalytic performance. This observation highlights the influence of Ni on the electronic properties of Pd and its interactions with CeO_2_. Electron donation from Ni to Pd may alter the electronic structure, promoting a more favorable environment for catalytic processes and possibly enhancing the efficiency of the PdNi/CeO_2_ electrocatalyst in comparison to Pd/CeO_2_.

The adsorption energies of IPA calculated using eq 1 further clarifies the catalytic performance of these systems. Adsorption on pure Pd(111) and PdNi(111) is measured at −1.51 and −1.23 eV, respectively, indicating moderate interactions and this property of adsorption energy of IPA on Pt(111) and Pd(111) and Ni(111) was also observed in previous study.^77^ In contrast, the adsorption energies for the supported catalysts were slightly more negative, with Pd/CeO_2_ at −2.08 eV and PdNi/CeO_2_ at −1.82 eV. According to the d-band model, the stronger the adsorption energy (more negative *E*_ad_ value), correspond to the upward shift of the d-band center (less negative value). But in this work, it is not so. A similar finding has been reported by others^78,79^ and was attributed to the ability of the adsorbate to adsorb at different sites on the catalyst. It seems that this is what could be happening in our case too. This substantial increase in the adsorption energy for both supported systems suggests that the interaction with CeO_2_ significantly enhances the adsorption of the IPA on the modeled catalysts.

Pd/CeO_2_ exhibits the strongest interaction with IPA, suggesting the highest adsorption of IPA. However, in PdNi/CeO_2_ demonstrates a decrease in adsorption energy of IPA due to the presence of electron donation of Ni toward the Pd, leading to optimal adsorption energy for IPA on the catalyst surface. This indicates that the combination of alloying and support synergistically improves catalytic behavior. These findings emphasize the importance of manipulating the electronic structure to design efficient catalysts toward the oxidation of IPA.

Table 3 displays the adsorption energies of IPA on the electrocatalysts, along with the d-band center, and the distance between the oxygen atom of IPA and the top Pd atom of each modeled catalysts. The downshift of the d-band center in this PdNi/OLC (−1.23 eV) was associated with reduced IPA adsorption and for PdNi/CeO_2_/OLC (−1.82 eV) due to the presence of Ni.

In addition to the d-band center, the d-bandwidth also plays a crucial role in determining adsorption strength. A narrower d-band corresponds to more localized d-states, which can enhance the interaction with adsorbate orbitals and result in stronger adsorption. This is particularly evident in the Pd(111)/CeO_2_ system, which exhibits a narrower d-band and the strongest adsorption energy among all configurations. A similar trend is observed for PdNi(111)/CeO_2_, where the d-band also narrows relative to the unsupported surface, contributing to stronger binding. These differences in d-band shape are clearly illustrated in Figure 10, which shows the PDOS for the Pd d-orbitals. The narrowing is likely due to strong metal–support interactions and electronic effects induced by the CeO_2_ support. These findings highlight that both the position and width of the d-band are important electronic descriptors for understanding adsorption behavior, particularly in supported or alloyed catalysts.

These findings highlight the crucial role of electronic structure manipulation, where the combination of PdNi alloying and CeO_2_ support optimizes IPA adsorption and enhances catalytic activity, with PdNi/CeO_2_ showing the most favorable adsorption energy and improved reactivity. This confirms both theoretically and experimentally that the synergy between alloying and support significantly enhance the electrocatalytic performance for IPA oxidation.

#### Proposed Reaction Mechanism

In alkaline conditions, IPA is oxidized to acetone via a stepwise dehydrogenation mechanism involving hydroxide ions (OH^–^) and active Pd sites. Note that key role of the metallic support (Ni/CeO_2_) as observed from physicochemical characterization and DFT simulation was to modulate the electronic structure of the Pd to enhance the electrocatalytic oxidation of IPA.

1. Adsorption process

- IPA adsorbs onto the Pd surface via its hydroxyl group. This adsorption is supported by our DFT calculations (see Figure 9)­
$${\left({\mathrm{CH}}_{3}\right)}_{2}\mathrm{CHOH}+{\;}^{*}\to {\left({\mathrm{CH}}_{3}\right)}_{2}{\mathrm{CHOH}}^{*}\left({\;}^{*}\;\mathrm{denotes}\;\mathrm{an}\;\mathrm{active}\;\mathrm{Pd}\;\mathrm{site}\right)$$
- Simultaneously, OH^–^ from the alkaline electrolyte adsorbs on the Pd and supporting catalyst active sites (as evident from electrocatalysis and previous studies)
$${\mathrm{OH}}^{-}+{\;}^{*}\to {\mathrm{OH}}^{*}+e^{-}$$

2. Dehydrogenation process:

- The OH* abstracts proton from the hydroxyl group to generate isopropoxide intermediate and water molecule:
$${\left({\mathrm{CH}}_{3}\right)}_{2}{\mathrm{CHOH}}^{*}+{\mathrm{OH}}^{*}\to {\left({\mathrm{CH}}_{3}\right)}_{2}{\mathrm{CHO}}^{*}+H_{2}O+e^{-}+{\;}^{*}$$

3. Removal of α-hydrogen (C–H)

- The second OH* abstracts hydrogen from the central carbon (α-position):
$${\left({\mathrm{CH}}_{3}\right)}_{2}{\mathrm{CHO}}^{*}+{\mathrm{OH}}^{*}\to {\left({\mathrm{CH}}_{3}\right)}_{2}{\mathrm{CO}}^{*}+H_{2}O+e^{-}+{\;}^{*}$$

4. Desorption of acetone:

- Acetone desorbs from the surface, regenerating the Pd active site
$${\left({\mathrm{CH}}_{3}\right)}_{2}{\mathrm{CO}}^{*}\to {\left({\mathrm{CH}}_{3}\right)}_{2}\mathrm{CO}+{\;}^{*}$$
$$\mathrm{overall reaction:}\;\mathrm{Pd-}{\left({\mathrm{CH}}_{3}\right)}_{2}{\mathrm{CHOH}}_{\left(\mathrm{ads}\right)}+\mathrm{Pd}{\mathrm{-OH}}_{\left(\mathrm{ads}\right)}\to {\left({\mathrm{CH}}_{3}\right)}_{2}\mathrm{CO}+2H_{2}O+2e^{-}+2\mathrm{Pd}$$

## Conclusions

In conclusion, this study investigates the electrocatalytic performance of Pd-based catalysts, including PdNi/OLC, Pd/CeO_2_/OLC, and PdNi/CeO_2_/OLC, for IPA oxidation reaction for fuel cell application. Our findings demonstrate that the incorporation of Ni and CeO_2_ significantly enhances the electrochemical properties and catalytic activity of Pd. Among the catalysts tested, PdNi/CeO_2_/OLC exhibited the highest ECSA and the most favorable electrochemical behavior, exhibiting improved IPA electrooxidation, and positive shifts in anodic peak potentials. When assessing the catalytic performance, PdNi/CeO_2_/OLC outperformed the other materials, displaying the highest peak current density 2017.3 mA/mg_Pd_ and low onset potential −0.69 V, indicative of superior reaction kinetics. EIS revealed a significantly *R*_ct_, suggesting a more efficient electron transfer. Additionally, chronoamperometric tests confirmed the high stability and antipoisoning capabilities of PdNi/CeO_2_/OLC, which maintained stable current densities over extended operation times, while the other catalysts suffered rapid current decay due to intermediate poisoning. DFT calculations provided further insights into the electronic structure of the catalyst, revealing that Ni incorporation into Pd enhances electron donation, which is further optimized by the CeO_2_ support. The synergy between Ni and CeO_2_ in PdNi/CeO_2_/OLC promoted IPA adsorption and improved the catalytic reactivity, reinforcing the experimental observations. These results emphasize the importance of alloying Pd with Ni and supporting it on CeO_2_ to enhance electrocatalytic activity, stability, and mass transport, making PdNi/CeO_2_/OLC a promising candidate for application in DIFC. Overall, the findings underscore the critical role of tailoring the electronic structure through alloying and support materials to optimize the electrocatalytic performance.

## Supplementary Material



## Notes

Additional experimental information, electrochemical measurements, synthesis of Pd/OLC, SEM EDX and HR-TEM EDX of PdNi/OLC, Pd/CeO_2_/OLC and PdNi/CeO_2_/OLC; wide survey XPS peaks of the electrocatalysts; C 1s spectra of XPS; electrochemical stability study (500 cycles at 100 mV/s) for the synthesized electrocatalysts; scan rate studies; adsorption energy vs d-band center of the electrocatalysts; thermogravimetric analysis (TGA) and derivative thermogravimetry (DTG); diffraction planes of Pd and 2θ values in the as-prepared electro-catalysts from the XRD experiments; crystallite size and lattice parameters of the synthesized electrocatalysts, EIS data and proposed reaction mechanism of oxidation of IPA to acetone in alkaline medium (PDF)
