# Supplementary material for: Electrocatalytic Properties and DFT Studies of Pd-Based Catalysts Supported on Ceria/Onion-like Carbon for Isopropanol Oxidation in Alkaline Medium
Source: J Phys Chem C Nanomater Interfaces. 2025 Jul 23;129(31):14214–28. doi: 10.1021/acs.jpcc.5c03686 (PMC12337145; doi:10.1021/acs.jpcc.5c03686)
Supplement: Supplementary file 1 [file jp5c03686_si_001.pdf]

SUPPORTING INFORMATION

Electrocatalytic Properties and DFT Studies of Pd-Based Catalysts Supported on Ceria/Onion-Like Carbon for Isopropanol Oxidation in Alkaline Medium

Desalegn Nigatu Gemechu[1,3], Aderemi B. Haruna[3], Ahmed Mustefa Mohammed[1], Yedilfana Setarge Mekonnen[2,*], and Kenneth I. Ozoemena[3,*]

[1.] *Department of Chemistry, College of Natural and Computational Sciences, Addis Ababa University, P. O. Box 1176, Addis Ababa, Ethiopia,*

[2.] *Center for Environmental and Computational Sciences, Addis Ababa University, 1176 Addis Ababa, Ethiopia*

[3.] *Molecular Science Institute, School of Chemistry, University of the Witwatersrand, Johannesburg 2050, South Africa*

[*]Authors to whom correspondence should be addressed: Yedilfana Setarge (e-mail: yedilfana.setarge@aau.edu.et) and K.I. Ozoemena (e-mail: Kenneth.ozoemena@wits.ac.za).

Electrochemical measurements

For preparing the slurry, 1 mg of the prepared catalyst was dispersed in a mixed solution of 5 μL Nafion (5 wt%) and 460 μL isopropanol alcohol and sonicated for 1 hr. For catalysing isopropanol oxidation reaction, 10 μL of the slurry was dropped onto a polished glassy carbon electrode with the catalyst loading of 0.002 mg and was used as the working electrode. A Pt electrode was used as the counter electrode and Ag/AgCl electrode was used as a reference electrode.

Synthesis of Pd/OLC

The synthesis commenced with the ultrasonication of 80 mg of OLC in 100 ml of ethylene glycol within a three-neck round-bottomed flask for 1 h. Following this, a solution consisting of 10 mL of H_2O, 10 mL of ethylene glycol, and 2 mL of 35% HCl, containing 31.3 mg of dissolved K_2PdCl_4, along with another solution containing 10 ml of ethylene glycol, 10 mL of water, was added dropwise under stirring in a nitrogen stream to remove dissolved oxygen. After thorough stirring, an alkaline solution of NaOH (1 g) in 10 mL of H_2O and 35 mL of ethylene glycol was introduced into the reactor, which was then heated at 125 °C for 3 h under a nitrogen atmosphere. Subsequently, the mixture was cooled to room temperature. The resulting solid product was filtered and washed with H_2O until reaching a neutral pH. Finally, the product was dried in a vacuum oven at 40 °C, yielding 90.8 mg of the desired product.

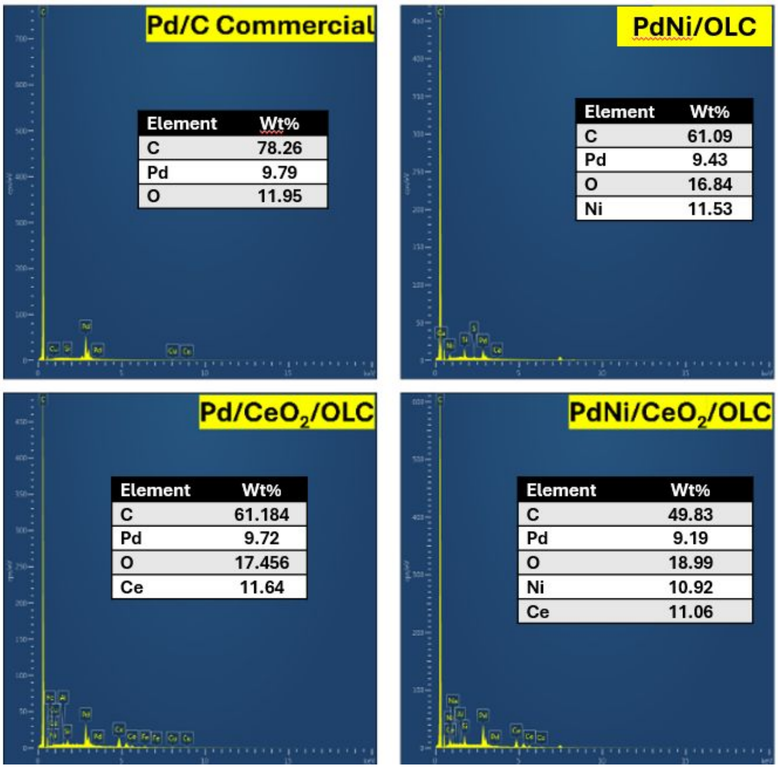

Figure S1: EDX spectrum of PdNi/OLC, Pd/CeO_2/OLC and PdNi/CeO_2/OLC

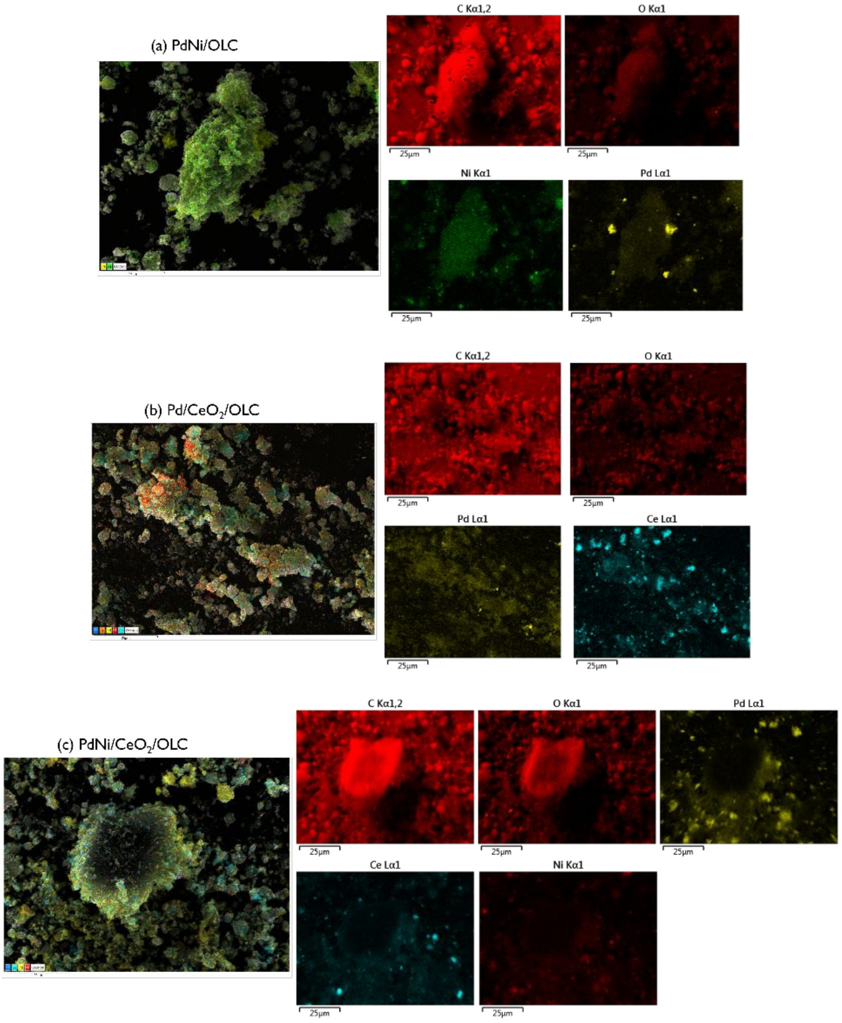

Figure S2: SEM EDX of PdNi/OLC, $Pd/CeO_2/OLC$ and $PdNi/CeO_2/OLC$

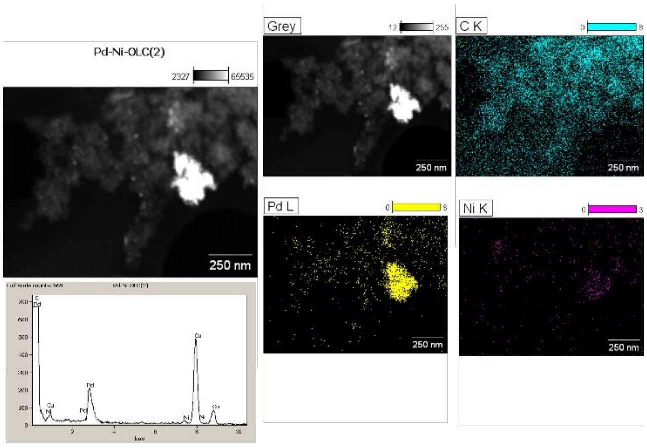

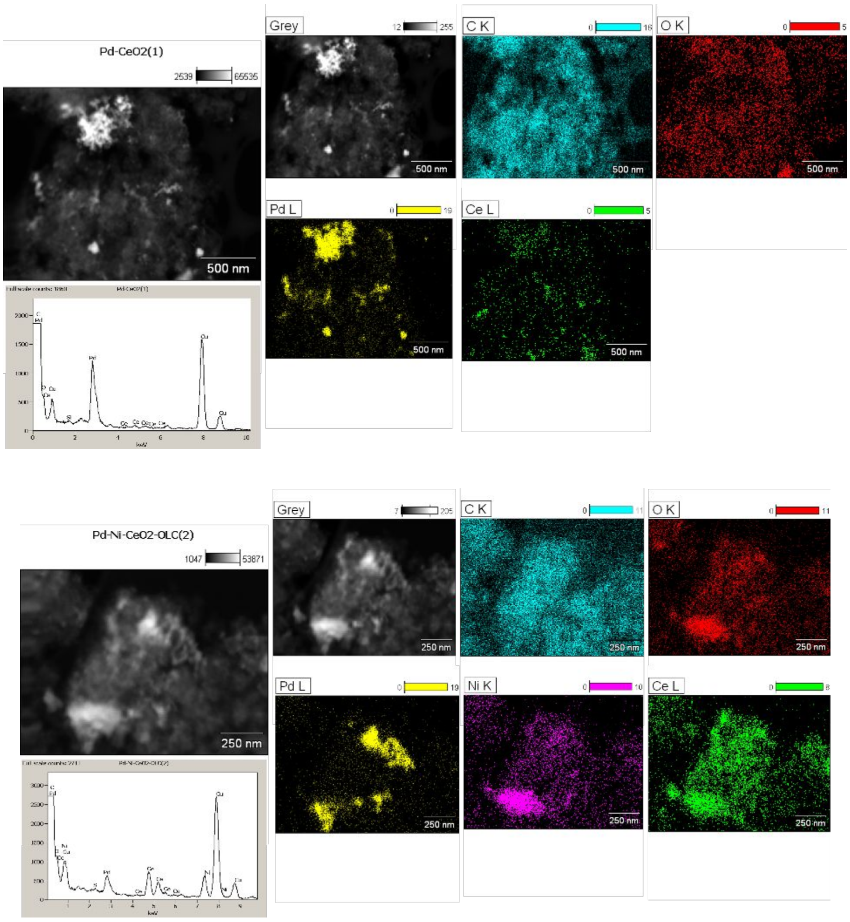

Figure S3: HR-TEM EDX of PdNi/OLC, Pd/CeO_2/OLC and PdNi/CeO_2/OLC

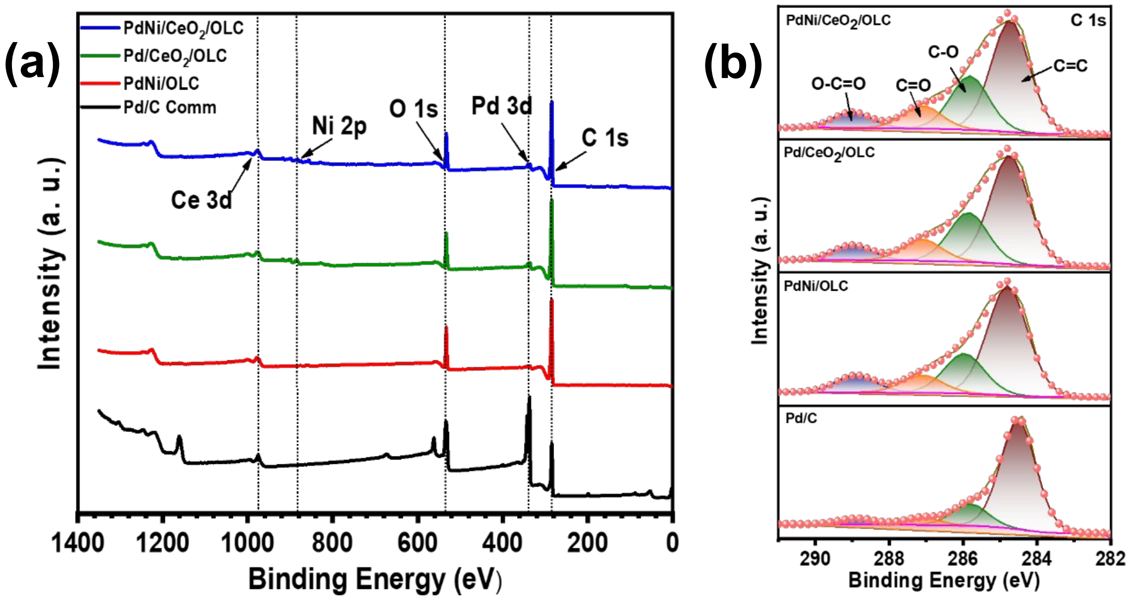

Figure S4: (a) wide survey XPS peaks for the electrocatalysts (b) C 1s spectra for the electrocatalysts

Stability study foe 500 cycles using 100mV/s for the synthesized electrocatalysts

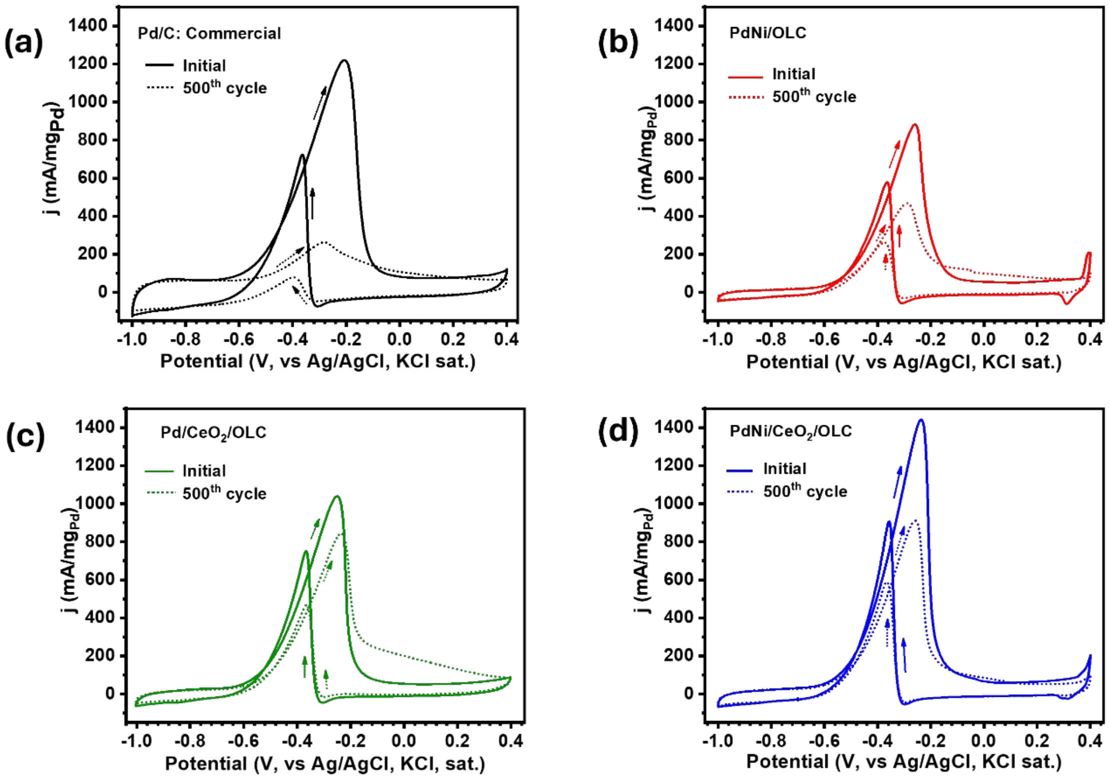

Figure S5: Stability study for the electrocatalysts for 500 cycles in 100 mv/s scan rate.

Scan rate study for the electrocatalysts starting from 20 mV/s up to 200 mV/s with the interval of 20 mV/s

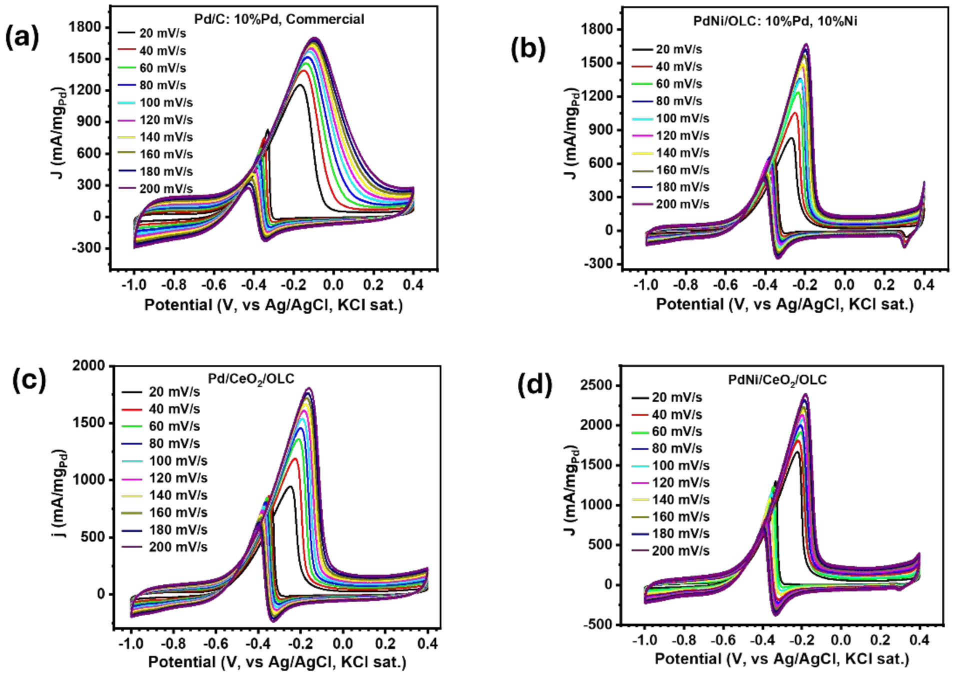

Figure S6: Scan rate study for the electrocatalysts

Adsorption energy vs d-band center of the electrocatalysts

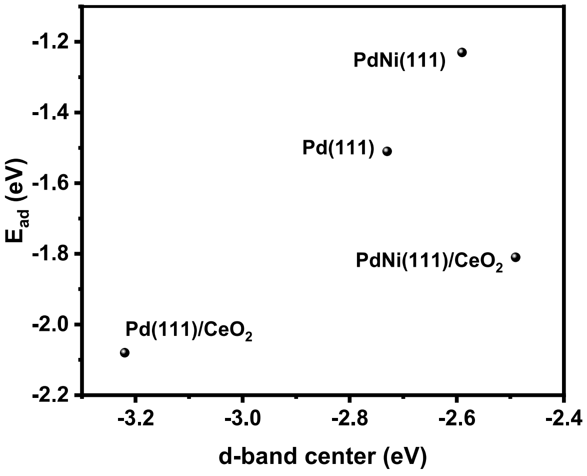

Figure S7: d-band center vs E_{ad} of the electrocatalysts

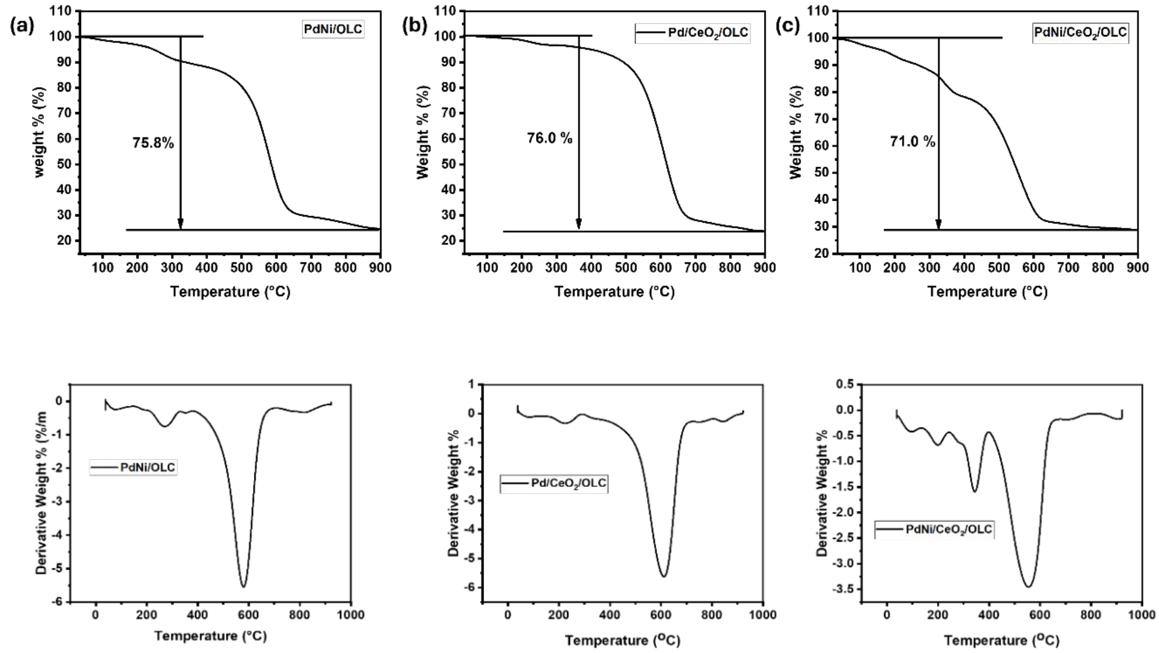

Figure S8: Thermogravimetric analysis (TGA) and derivative thermogravimetry (DTG) for the (a)PdNi/OLC, (b) $Pd/CeO_2/OLC$ and (c)$PdNi/CeO_2/OLC$

Table S1: The diffraction planes of Pd and 2θ values in the as-prepared electro-catalysts from the XRD experiments.

Catalysts	Diffraction planes and 2θ (degree)		
	(111)	(200)	(220)
Pd/OLC	40.08	46.59	68.15
PdNi/OLC	39.55	46.35	67.49
$Pd/CeO_2/OLC$	39.39	46.19	67.17
$PdNi/CeO_2/OLC$	39.24	46.16	67.12

Table S2: Crystallite size and lattice parameters of the synthesized electrocatalysts

**Catalyst**	**Crystalite size (nm)**	**Lattice parameter (Å)**
PdNi/OLC	4.9	3.93
$Pd/CeO_2/OLC$	5.1	3.95
$PdNi/CeO_2/OLC$	4.7	3.98

Table S3: EIS parameters

**Catalysts**	**R_s**	**CPE (×10^{-3})**	**a**	**R_{ct}**
Pd/C	46.77±0.97	1.72±0.11	0.65±0.002	181.3+8.48
PdNi/OLC	29.43±0.10	0.71±0.02	0.86±0.006	163.3+2.35
Pd/CeO_2/OLC	38.85±0.21	0.74±0.01	0.86±0.005	251.3±3.81
PdNi/CeO_2/OLC	28.90±0.12	0.75±0.01	0.86±0.004	142.4±0.99
